# The effect of transformation policies on healthcare providers’ satisfaction in primary healthcare centers: the case of Eastern Saudi Arabia

**DOI:** 10.1186/s12913-023-10335-8

**Published:** 2023-11-30

**Authors:** Arwa Abdulrahman Althumairi, Fatmah Muhammad Bukhari, Layan Bassam Awary, Duaa Aljabri

**Affiliations:** Department of Health Information Management and Technology, College of Public Health, Imam Abdulrahman bin Faisal University, P.O. Box 2954, Dammam, 6603-34211 Saudi Arabia

**Keywords:** Job satisfaction, Primary HealthCare (PHC), Patient-centered care, Quality of care, Health Sector Transformation Program (HSTP)

## Abstract

**Background:**

The Saudi Arabian Vision 2030 encompasses the Health Sector Transformation Program (HSTP), an initiative aimed at enhancing the accessibility, affordability, and quality of healthcare, with a strong emphasis on patient-centered care. To achieve this vision, the government has been providing training to Primary Healthcare (PHC) centers on patient-centered care, recognizing that spending quality time with patients is crucial for making informed clinical decisions. Therefore, it is essential to evaluate provider satisfaction with the quality of services they provide and assess the impact of organizational factors on care quality. This study represents the first comprehensive assessment of job satisfaction among PHC providers in the Eastern region of Saudi Arabia. It seeks to gauge job satisfaction among PHC providers and explore its associated impact on the quality of care they deliver.

**Methods:**

This study employed a quantitative cross-sectional design. Data were collected using a modified version of the Job Satisfaction Survey (JSS), supplemented by three newly added dimensions. Additionally, questions addressing general characteristics were incorporated into the survey instrument. Data analysis involved calculating frequencies and percentages for univariate analysis, employing t-tests for comparisons between two groups, and utilizing ANOVA for comparisons among multiple groups (bivariate analysis).

**Results:**

A total of 143 PHC providers took part in this study. Of these, 48% reported high satisfaction, while the rest were either dissatisfied or neutral. PHC providers were highly satisfied with supervision (17%, *N*=94). On the other hand, they were dissatisfied with contingent rewards (3%, *N*=15). There was a significant difference found between the intention to leave the job (yes, no) and job satisfaction scores (mean (SD)= 83.58 (16.174) vs. mean (SD)=101.64 (16.209), *p*-value < 0.001). There were also significant relationships between general characteristics and the dimensions such as co-workers, promotion, responsibility, nature of work, operating procedure, and communication (*p*-value< 0.05).

**Conclusion:**

The main findings of this study suggest that PHC providers working in PHC centers in the Eastern region were satisfied with their work, especially with supervision and patient care. However, the findings also revealed that there are many areas of the job of PHC providers that require planned reform, such as contingent reward and communication. Furthermore, intention to leave the job was significantly related to job satisfaction score and all the dimensions. The study findings will help policymakers and the Ministry of Health to develop an employee engagement and satisfaction program to track the PHC providers' levels of satisfaction.

**Supplementary Information:**

The online version contains supplementary material available at 10.1186/s12913-023-10335-8.

## Introduction

Primary HealthCare (PHC) centers are important because they are the first point of interaction between community members and healthcare services [1, 2]. The Saudi Arabian 2030 vision includes the Health Sector Transformation Program (HSTP), which is designed to improve healthcare accessibility, affordability and quality while emphasizing patient-centered care [3]. To achieve this, the government is providing training to PHC centers on patient-centered care, as time spent with patients is key for proper clinical decisions. Therefore, assessing provider satisfaction with the quality of services they provide and the impact of the organization on care quality is important [4, 5].

Job satisfaction is “the feeling of pleasure and achievement that you experience in your job when you know that your work is worth doing or the degree to which your work gives you this feeling” [6]. There are number of reasons why job satisfaction is important in the work environment, this is because of high job satisfaction level will result in increase productivity, increase employee loyalty, increase customer satisfaction, and decrease employee turnover [6–9]. Few studies assessed the job satisfaction among healthcare providers working in PHC centers in Saudi Arabia. Most of these studies were conducted in different geographical areas in the Kingdom of Saudi Arabia: Riyadh, Jazan, Jeddah, and a comparison between Jeddah and Eastern region. The literatures were conducted from 1999 to 2020, this era demonstrated the change in job satisfaction of physicians and nurses in PHC centers in the Kingdom of Saudi Arabia. Each study was concerned with measuring the job satisfaction level of physicians and/or nurses by using online questionnaires as a data collection method [10–12].

Several studies identified the factors affecting PHC providers’ job satisfaction working in PHC centers. These studies were mainly for physicians and/or nurses. They found that most factors influencing physicians’ job dissatisfaction were lack of incentives, especially financial, lack of essential medical equipment, and lack of administrative support. Additionally, the community and other physicians with different specialists consider family physicians as an inferior specialty[7, 13]. While for nurses was poor staffing, management practices, low salary and financial incentives, low improvement opportunities, and lack of care supplies [10]. These studies reveal that physicians and nurses face obstacles in their working life. Therefore, these incentives that lead to decreasing job satisfaction levels should be addressed and resolved. On the other hand, the factors that affect physicians’ and nurses’ job satisfaction. For physicians, job satisfaction increases when working within teams, such as taking place with mass vaccination, providing health education, social support, infection control, and environmental health. Moreover, it was also found that the highest level of satisfaction was noticed toward the nature of work, freedom to make the clinical decision, spending more time with patients, and not having additional administrative work [10, 14]. While, for nurses, was the influence of their co-workers [14]. Therefore, these incentives that help in increasing job satisfaction levels should be encouraged and promoted.

Each study was concerned with evaluating the job satisfaction level of physicians and/or nurses used an online questionnaire as a data collection method [14–16]. The questionnaire consists of two parts; the first concerns the socio-demographic data, and the second contains questions about the possible factors that could affect physicians’ job satisfaction. Accordingly, this study seeks to gauge job satisfaction among PHC providers and explore its associated impact on the quality of care they provide. Specifically, it aims to ascertain the factors which influence job satisfaction, and the correlation between job satisfaction and the likelihood of leaving one’s job. In addition, this study used a modified version of the JSS. There were eight dimensions used from JSS, which were: payment, promotion, supervision, operating procedures, contingent rewards, co-workers, communication, nature of work. In this study there were three newly added dimensions, which were: responsibility, patient care, and social life. These new dimensions were used to measure their impact on job satisfaction.

## Methods

### Study population

The study was undertaken in PHC centers in the Eastern region of Saudi Arabia using quantitative cross-sectional study. The target population was PHC providers (physicians, nurses, allied healthcare workers, and public health professionals) working in PHC centers in the Eastern region of Saudi Arabia. Administration staff were excluded from the study, as they do not provide direct healthcare services to patients.

### Sample size

The sample was recruited using a convenience sampling strategy. The study’s sample size was calculated using the following formula: *n* = Z^2 * p * q / d^2, where n is the sample size, Z is the z-score for a 95% confidence level (1.96), p is the estimated proportion of PHC providers who are likely to meet the inclusion criteria (50%), q is 1-p, and d is the margin of error (10%). This calculation resulted in a minimum sample size of 94 PHC providers. This study reached 143 PHC providers who actually provide healthcare services to patients. The study’s sample size was calculated using the Calculator.net website [17], the population size of PHC providers in the Eastern region was 3,611.

### Data collection methods

The study’s questionnaire was consists of three sections, the first section was an introduction to the questionnaire that contains the study objective, study setting, target population, and the researchers’ contact information (Supplementary Table S2). As well as an explicit consent statement. The second section was adapted from JSS with few modifications. There were eight dimensions used from JSS, which were: payment, promotion, supervision, operating procedures, contingent rewards, co-workers, communication, nature of work, and in this study, there were three newly added dimensions which were: responsibility, patient care, and social life. The JSS is a well-established instrument that had been repeatedly investigated for reliability and validity [11]. The participant responses were collected using 5-domain Likert scale ranging from “strongly agree” to “strongly disagree” as follows: [5] strongly agree, [4] agree, [3] neutral, [2] disagree, and [1] strongly disagree. The third section contains general questions which are gender, age, marital status, educational level, specialty, years of experience, salary range, travel time to the PHC center, shift period, working hours, thought of leaving the job during the last two years.

### Procedure and timeline

A web-based questionnaire was distributed to the participants through social media platforms such as LinkedIn, twitter, WhatsApp and emails. The data collection process was taken place from February 2023 to April 2023.

### Analysis

This study start with a descriptive analysis of the participant characteristics and satisfaction domains, using univariate analysis for frequencies, mean and percentages. In addition, bivariate analysis using t-test and ANOVA that was applied depend on the variable type after assessing study normality. Moreover, a multilinear regression model was applied to identify the factors that corelated with overall satisfaction score All of these tests were conducted through SPSS version 29, in 95% confidence interval level.

### Ethical considerations

Ethical approval from the Institutional Review Board at Imam Abdulrahman bin Faisal University, Dammam was obtained prior to the conduction of the study (IRB-UGS-2023-03-057). In addition to an explicit approval from participant to take part in the research and use their data for publication purposes. Written informed consent to participate in the study was obtained from all participants. All human procedures were performed in accordance with the guidelines of the Declaration of Helsinki of 1975.

## Results

### Sample profile

Out of 276 participant received the questionnaire only 143 (52%) completed the questionnaire. Table 1 showed that most of the respondents’ specialties were physicians (*n* = 60, 42%) followed by nurses (*n* = 57, 39.9%). The majority of respondents were female (*n* = 110, 76.9%), aged between 31 and 40 (*n* = 74, 51.7%), married (*n* = 105, 73.4%). Most of the respondents held a bachelor’s degree (*n* = 75, 52.4%), and received a monthly salary of 10,000 to 20,000 (*n* = 81, 56.6%), with years of experience equal to or more than 10 years (*n* = 80, 55.9%). Most of the respondents worked for 8 h (*n* = 141, 98.6%), in the morning shift (*n* = 131, 91.6%), and the time that is taken from home to PHC center is 16 to 35 min (*n* = 45, 31.5%). 45.5% (*n* = 65) thought of leaving their job in the past 2 years.

### Relation between general characteristics and perceived job satisfaction

Using bivariate analysis the relation between job satisfaction and general characteristics. No significant differences were found between perceived job satisfaction and the general characteristics, which were: gender, age, marital status, education level, specialty, years of experience, salary range, time taken from home to PHC centers, shift time, and working hours (*p*-value > 0.05, Table 1).

**Table 1:** Distribution of general characteristics with mean of the perceived job satisfaction score *N*=143

	N (%)	Mean (SD)	Test value	*P*-value
Gender				
Male	33 (23.1)	97.7 (20.7)	-1.528	0.129
Female	110 (76.9)	92.1 (17.7)		
Age				
21-30	33 (23.1)	96.1 (17.2)	1.492	0.229
31-40	74 (51.7)	90.9 (19.1)		
>41	36 (25.2)	96.3 (18.1)		
Marital status				
Single	27 (18.9)	94.5 (17.5)	0.140	0.869
Married	105 (73.4)	93.4 (19.2)		
Divorce / Widow	11 (7.7)	91.0 (15.0)		
Education Level				
Diploma degree	47 (32.9)	96.8 (16.1)	1.610	0.204
Bachelor’s degree	75 (52.4)	92.7 (20.2)		
Postgraduate (Master / PhD)	21 (14.7)	88.5 (16.5)		
Specialty				
Physician	60 (42.0)	94.6 (19.1)	1.767	0.156
Nurse	57 (39.9)	94.6 (15.6)		
Allied healthcare^a^	18 (12.6)	92.3 (18.9)		
Public health	8 (5.6)	79.3 (28.1)		
Years of experience				
<=1	14 (9.8)	90.1 (19.4)	0.193	0.901
2 – 5	24 (16.8)	92.9 (19.9)		
6 – 9	25 (17.5)	94.6 (21.3)		
>=10	80 (55.9)	93.8 (17.2)		
Salary range				
<10,000	26 (18.2)	92.0 (21.6)	0.245	0.783
10,000 – 20,000	81 (56.6)	94.4 (17.2)		
>20,000	36 (25.2)	92.3 (19.3)		
Time from home to PHC centers				
0 – 15	43 (30.1)	92.8 (20.5)	0.282	0.838
16 – 35	45 (31.5)	93.6 (19.7)		
36 – 55	34 (23.8)	92.0 (14.0)		
>55	21 (14.7)	96.6 (18.9)		
Shift Time				
Morning	131 (91.6)	93.5 (18.0)	0.454	0.636
Afternoon	3 (2.1)	102.0 (30.1)		
Evening	9 (6.3)	90.2 (23.1)		
Working Hour				
8 hours	141 (98.6)	93.5 (18.3)	0.533	0.595
12 hours	2 (1.4)	86.5 (36.1)		

^a^Lab technician, Radiologist, Pharmacist

### Relation between general characteristics and job satisfaction dimensions

Tables s1 in the supplementary section represented bivariate analysis results between general characteristics and job satisfaction’s dimensions.

Based on the post hoc analysis physicians had significant higher satisfaction score of promotion dimension compared with allied health and public health specialist (Mean = 6.68 vs. 5.11 and 4.38 respectively, *p* = 0.002, Table Supplementary S1).

PHC providers with diploma degree had significantly higher satisfaction score of operating procedures dimension compared with PHC providers with bachelor’s degree (Mean = 6.96 vs. 5.85 respectively, *p* = 0.01, Table Supplementary S1).

Single PHC providers had a significantly higher satisfaction score of co-workers dimension compared with divorce/ widow PHC providers (Mean = 10.22 vs. 8.36 respectively, *p* = 0.045). Physicians had significantly a higher satisfaction score of co-workers dimension compared with nurses (Mean = 10.03 vs. 8.79 respectively, *p* = 0.012). PHC providers who work < = 1 year have significant higher satisfaction score of co-workers dimension compared with PHC providers who work > = 10 years (Mean = 10.71 vs. 9.08 respectively, *p* = 0.02, Table Supplementary S1).

PHC providers with diploma degree had significantly a higher satisfaction score in communication dimension compared with PHC providers with postgraduate (master/ PhD)(Mean = 3.77 vs. 2.9 respectively, *p* = 0.011*, Table Supplementary S1).

PHC providers who work 8 h a day had significantly higher satisfaction score of responsibility dimension compared with PHC providers who work 12 h a day (Mean = 8.04 vs. 5.5 respectively, *p* = 0.007*, Table Supplementary S1).

Patient care dimension, social life, supervision dimension and contingent rewards dimension were not statistically significant with participants’ general characteristics.

### Intention to leave the work

Table 2 showed that there was an overall a significant difference found between intention to leave (yes, no) the job and job satisfaction scores (mean (SD) = 83.58 (16.174) vs. mean = 101.64 (16.209) respectively, *p*-value < 0.001**). This significant relationship was present for all the dimensions.

**Table 2:** The relation between job satisfactions dimensions and intention to leave the job

Dimensions	Yes ***n***=65	No ***n***=78	Test value	***P***-value
	mean (SD)	mean (SD)		
Pay dimension	5.6 (2.1)	6.7 (1.7)	-3.564(141)	0.001
Promotion dimension	5.3 (2.0)	6.6 (2.0)	-3.885(141)	<0.001
Supervision dimension	13.7 (3.8)	18.0 (4.0)	-6.552(141)	<0.001
Contingent rewards	2.7 (1.1)	3.5 (1.1)	-4.564(141)	<0.001
Operating procedures	5.1 (1.7)	7.1 (2.1)	-6.245(141)	<0.001
Co-workers	9.0 (2.5)	9.8 (2.0)	-2.192(141)	0.030
Nature of work	13.7 (3.6)	17.4 (2.7)	-6.748	<0.001
communication	3.1 (1.2)	3.7 (1.0)	-3.497	0.001
Responsibility	7.7 (1.4)	8.2 (1.2)	-2.189(141)	0.030
Patient care	12.4 (3.1)	13.8 (3.5)	-2.496(141)	0.014
Social life	5.3 (2.0)	6.9 (1.8)	-4.948	<0.001
Total job satisfaction	83.6 (16.2)	101.6 (16.2)	-6.640	<0.001

### Overall job satisfaction score

Table 3 showed that the overall PHC providers’ job satisfaction was 48% (strongly agree and agree) compared to 27% (strongly disagree and disagree). PHC providers were highly satisfied with supervision 17%. *N* = 94. On the other hand, PHC providers were dissatisfied with contingent rewards (20%, *N* = 85). There was strong correlation between perceived and actual satisfaction scored, the more the employee rank the perceived satisfaction was satisfied they were more likely to be actually satisfied with their work. This was more likely offered to the supervisor dimension (with mean of satisfaction 10 when they are actually disagree compared with mean of 20 when they actually agree).

**Table 3:** Distributions of participant agreement level and job satisfaction domain

Perceived satisfaction dimensions	Actual satisfactions					F value	***P*** value
	Strongly Disagree	Disagree	Neutral	Agree	Strongly Agree		
	***N***=13	***N***=12	***N***=33	***N***=64	***N***=21		
	Mean(SD)	Mean(SD)	Mean(SD)	Mean(SD)	Mean(SD)		
Payment	4.3 (2.2)	6.6 (1.5)	5.6 (1.8)	6.6 (1.7)	7.1 (2.1)	6.372	<.001
Promotion	4.2 (1.6)	5.4 (1.8)	5.9 (2.0)	6.1 (1.9)	7.5 (2.5)	5.913	<.001
Supervision	9.6 (2.9)	12.5 (4.0)	15.0(3.4)	17.3 (3.6)	19.8 (3.3)	22.939	<.001
Rewards	1.6 (0.6)	1.8 (1.0)	3.0 (1.0)	3.6 (0.8)	3.8 (1.1)	20.465	<.001
Procedures	3.9 (1.5)	4.3 (1.3)	5.6 (1.6)	6.9 (1.8)	7.8 (2.0)	17.814	<.001
Coworkers	8.4 (3.5)	8.6 (1.8)	9.0 (1.8)	9.9 (2.1)	9.7 (2.3)	2.386	0.054
Communication	2.5 (1.5)	2.6 (1.0)	3.4 (1.0)	3.6 (1.0)	4.1 (1.1)	7.135	<.001
Responsibility	7.2 (1.8)	7.9 (1.3)	7.4 (1.1)	8.2 (1.2)	8.9 (1.1)	6.321	<.001
Patient care	7.3 (3.2)	12.8(1.8)	12.6 (2.2)	14.1 (2.8)	15.1 (3.2)	20.54	<.001
Social relationship	3.1 (1.3)	3.8(1.7)	5.7 (1.6)	6.9 (1.4)	7.8 (1.8)	31.01	<.001
Nature of work	9.5 (2.8)	11.8 (3.3)	14.4 (2.0)	17.4 (2.0)	18.8 (2.3)	55.457	<.001
Total perceived satisfaction	61.5 (12.5)	78.0(11.8)	87.4(11.1)	100.4(12.5)	110.2(15.8)	41.11	<.001

Multilinear regression model showed a statistical significant relation between specialty, participant from public health have lower satisfaction score compared with physicians (*p* value 0.016, Table 4). Additionally, years of experience had a significant relation with satisfaction (6–9 year have significant higher satisfaction score compared with less than 1 year, *p* value 0.03). Participant with intention to leave job have a significant lower satisfaction score compared with participant with no intention to leave their job at PHC centers.

**Table 4:** Multivariable linear regression model between total perceived satisfaction score and related factors

General characteristics	Beta	Test value	***P*** value	Confidence Interval	
				Lower Bound	Upper Bound
**Gender**					
Male	1.00 (ref)				
Female	0.072	0.886	0.378	-3.904	10.226
**Age**					
21-30	1.00 (ref)				
31-40	0.28	1.88	0.062	-0.645	25.171
>41	-0.13	-1.32	0.189	-11.971	2.394
**Marital status**					
Single	1.00 (ref)				
Married	0.104	0.998	0.320	-4.262	12.934
Divorce / Widow	0.026	0.289	0.773	-10.642	14.286
**Education Level**					
Diploma degree	1.00 (ref)				
Bachelor’s degree	-0.105	-0.923	0.358	-12.206	4.444
Postgraduate (Master / PhD)	-0.044	-0.401	0.689	-13.506	8.952
**Specialty**					
Physician	1.00 (ref)				
Nurse	-0.027	-0.216	0.830	-10.441	8.39
Allied healthcare	-0.105	-1.174	0.242	-15.695	4.006
Public health	-0.209	-2.437	**0.016**	-30.343	-3.145
**Years of experience**					
<=1	1.00 (ref)				
2 – 5	0.113	0.847	0.399	-7.418	18.51
6 – 9	0.327	2.136	**0.035**	1.16	30.556
>=10	0.403	1.918	0.057	-0.475	30.362
**Salary range**					
<10,000	1.00 (ref)				
10,000 – 20,000	-0.062	-0.605	0.547	-9.837	5.234
>20,000	-0.024	-0.2	0.842	-11.216	9.161
**Shift Time**					
Morning	1.00 (ref)				
Afternoon	0.053	0.684	0.495	-12.828	26.368
Evening	-0.025	-0.319	0.751	-13.871	10.025
**Working Hour**					
8 hours	1.00 (ref)				
12 hours	-0.041	-0.528	0.598	-30.593	17.709
**Intention to leave**					
Yes	1.00 (ref)				
No	0.477	6.145	**<.001**	11.971	23.35

Bold font for statical significant value stated for a value less than 0.05 or 0.01

### Study limitations

This is the first study that assessed all PHC providers’ job satisfaction in PHC centers in the Eastern region. Due to the uniqueness of PHC providers, their work difficulties, and the long working hours, the response rate was 52%. This might reduce the generalizability of the study. However, this study exceed the minimum estimated sample size, which was 94 PHC providers. Additionally, this study focused only in one region, it would be better to conduct it nationally. The data collection method used in this study was a self-reporting questionnaire as most used methods to collect data based on our literature review. However, the self-reporting questionnaire leaves it up to the participants to interpret the questions. This may decrease the reliability of responses because of misinterpretation of some questions. Despite these limitations, the findings of the study provide an important contribution to the existing body of knowledge.

## Discussion

Measuring job satisfaction among PHC providers is important, as it may directly influence patient satisfaction and the quality of their health care. Additionally, high job satisfaction levels lead to increased productivity, employee loyalty, customer satisfaction, and decreased turnover.

The main finding in this study suggests that PHC providers working in PHC centers in the Eastern region were satisfied with their work. Especially, with supervision, patient care, nature of work, responsibility. This was different than in other regions where only one-third of the PHC providers were satisfied with their work [4, 18, 19]. It could be suggested that leadership have a strong influence in employee satisfaction [4, 20], and this domain have high satisfaction score among the study population.

Despite the overall high levels of satisfaction reported by PHC providers in our study, several areas for improvement were identified, including contingent reward, communication, operating procedures, and promotion. These findings are consistent with national and international studies, which have also found that reward and promotion are essential elements of physician and nurse satisfaction, particularly in emotionally and physically demanding environments such as patient-centered care [4, 14, 21].

In our study, we could not find any relationship between general characteristics such as gender and age group and job satisfaction. Similarly, in the literature, gender, age groups, nationality, or marital status, have no relation with satisfaction sore [4, 19]. While other sociodemographic factors, have potential positive relationship with satisfactions these were years of experience, and specialty [22, 23]. Work-related difference might be the leading factors for employment satisfaction thus work stability.

In our study we measure job satisfaction by eleven dimensions and there were six dimensions that have a significant relation with the general characteristics. These are, the co-workers dimension has three influencing factors. Starting with years of experience the study found that PHC providers who work less than one year have a highest satisfaction mean score compared with those who worked 6 to 9 year or more than 10 years. Furthermore, single PHC providers have the highest satisfaction mean score compared with married and divorced or widow PHC providers, as supported by the literature [22].

Our study discovered that physicians reported high job satisfaction due to the collaborative teamwork they experienced in PHC centers, which provided them with valuable support from their co-workers. This corroborates previous findings; It was found that nurses in PHC centers were satisfied due to mutual respect and communication from physicians, as well as the friendships they developed [14]. Additionally, physicians working in PHC centers in Switzerland reported satisfaction due to the supportive work environment [12, 15].

Most respondents in the present study were satisfied with rewards and promotions because their efforts were acknowledged, and they had the chance for promotion. However, other studies suggest that most physicians in PHC centers were dissatisfied with rewards and fringe benefits, due to difficulty in obtaining financial incentives [11]. It was found Chinese PHC physicians were dissatisfied with job reward and perceived lack of career development or promotion in technical titles [24]. Therefore, though our respondents were satisfied with promotion potential, further effort is needed to monitor employee satisfaction.

Regarding our study, we measured some new dimensions such as responsibility and operating procedures. The study found that allied healthcare specialties were more satisfied with the policies and procedures in the PHC centers which influence the way they carry out their job tasks in better way. Alongside, physicians were satisfied with their responsibility for their work, and they feel that their role in PHC centers has an impact on public health prevention. This is can be justified that the quality of work in terms of rules and regulation is an important factor to attain employee satisfactions [25–28].

PHC providers were intending to leave their jobs have lower job satisfaction than those who stay, suggesting better patient care as there it was proved that there is a correlation between job satisfaction and patient satisfaction [9, 29]. In Jeddah and Riyadh it was confirm this, stating that maladapted physicians cannot provide optimal care [7, 30]. Patient-physician relationships can affect turnover rates, as they are an essential element in job satisfaction [31]. Primary healthcare workers are especially vulnerable to violence in the workplace and this can lead to job dissatisfaction and intention to leave [32]. Job satisfaction is essential for quality healthcare delivery in PHCs. That is why policy makers should measure job satisfaction regularly. In this study, PHC providers in Eastern region were generally satisfied with their job, although satisfaction differed depending on the specialty. Those who are more satisfied are less likely to leave, improving the quality of health care services.

## Conclusion

This study provides a comprehensive assessment of job satisfaction among PHC providers in the Eastern region. Our findings reveal that while providers generally expressed satisfaction with their work, there is room for improvement in several areas, including contingent rewards, communication, operating procedures, and promotion. These findings highlight the importance of addressing these concerns to enhance job satisfaction and potentially improve the quality of care provided in PHC centers.

Although this study offers valuable insights into job satisfaction among PHC providers, it is important to acknowledge its limitations. The study’s cross-sectional design limits our ability to establish causal relationships between job satisfaction and other factors. Additionally, the study’s reliance on self-reported data may introduce some bias. Future research should address these limitations by employing longitudinal designs and utilizing objective measures of job satisfaction and other variables of interest. Despite these limitations, our findings provide a foundation for further research and policy interventions aimed at enhancing job satisfaction among PHC providers. Addressing the identified areas of concern could contribute to improved quality of care, reduced provider turnover, and a more robust healthcare system in the Eastern region.

### Supplementary Information

**Additional file 1: Table S1.** Relation between PHC providers’ General characteristics and Mean score of some of the job satisfaction dimension. **Table S2.** Study survey questions.

**Additional file 2: Supplementary 2.** Database of Healthcare providers' job satisfaction in primary healthcare centers.

## Data Availability

All data generated or analyzed during this study are included in this published article.
