# Supplementary material for: The effect of transformation policies on healthcare providers’ satisfaction in primary healthcare centers: the case of Eastern Saudi Arabia
Source: BMC Health Serv Res. 2023 Nov 30;23:1328. doi: 10.1186/s12913-023-10335-8 (PMC10691092; doi:10.1186/s12913-023-10335-8)
Supplement: Supplementary file 1 — Additional file 1: Table S1. Relation between PHC providers’ General characteristics and Mean score of some of the job satisfaction dimension. Table S2. Study survey questions. [file 12913_2023_10335_MOESM1_ESM.docx]

Supplementary

Table s1:Relation between PHC providers’ General characteristics and Mean score of some of the job satisfaction dimension.

| **General characteristics** | **Promotion** | **Operating procedures** | **Co-workers** | **Nature of work** | **Communication** |
| --- | --- | --- | --- | --- | --- |
|  | **mean (SD)** | **mean (SD)** | **mean (SD)** | **mean (SD)** | **mean (SD)** |
| **Gender** |  |  |  |  | |
| Male | 6.2 (2.3) | 6.5 (2.2) | 10.0 (2.2) | 16.4 (3.4) | 3.6 (1.2) |
| Female | 6.0 (2.1) | 6.1 (2.1) | 9.2 (2.2) | 15.5 (3.6) | 3.4 (1.1) |
| **Age** |  |  |  |  |  |
| 21-30 | 6.1 (2.0) | 6.4 (1.7) | 10.0 (2.1) | 16.2 (3.7) | 3.6 (0.9) |
| 31-40 | 6.0 (2.2) | 5.9 (2.2) | 9.2 (2.3) | 15.1 (3.6) | 3.2 (1.2) |
| >41 | 5.9 (2.2) | 6.6 (2.3) | 9.4 (2.1) | 16.6 (3.3) | 3.7 (1.0) |
| **Marital status** |  |  |  |  |  |
| Single | 5.4 (2.1) | 6.4 (1.7) | **10.2 (2.6)** | 15.9 (3.8) | 3.6 (1.1) |
| Married | 6.2 (2.1) | 6.2 (2.2) | **9.3 (2.1)** | 15.6 (3.7) | 3.4 (1.2) |
| Divorce / Widow | 5.6 (2.0) | 5.8 (2.1) | **8.4 (1.7)** | 16.8 (2.6) | 3.3 (0.9) |
| **Education Level** |  |  |  |  |  |
| Diploma degree | 5.9 (2.3) | **7.0 (1.9)** | 8.8 (2.4) | **16.7 (3.1)** | **3.8 (0.8)** |
| Bachelor’s degree | 6.2 (2.1) | **5.9 (2.1)** | 9.7 (2.2) | **15.4 (3.8)** | **3.4 (1.2)** |
| Postgraduate (Master / PhD) | 5.7 (1.9) | **5.8 (2.1)** | 9.7 (1.9) | **14.7 (3.5)** | **2.9 (1.3)** |
| **Specialty** |  |  |  |  |  |
| Physician | **6.7 (1.9)** | 5.9 (2.2) | **10.0 (2.2)** | 15.4 (3.8) | 3.3 (1.2) |
| Nurse | **5.8 (2.3)** | 6.5 (1.8) | **8.8 (2.1)** | 16.5 (3.1) | 3.6 (1.01) |
| Allied healthcare | **5.1 (2.3)** | 6.8 (2.3) | **9.7 (2.3)** | 15.3 (3.2) | 3.7 (1.1) |
| Public health | **4.4 (1.3)** | 5.1 (2.9) | **8.5 (2.1)** | 13.1 (5.7) | 3.0 (1.3) |
| **Years of experience** |  |  |  |  |  |
| <=1 | 5.6 (1.7) | 6.3 (1.7) | **10.7 (2.2)** | 14.8 (4.3) | 3.6 (0.6) |
| 2 – 5 | 6.4 (2.1) | 6.0 (2.0) | **9.0 (2.5)** | 15.7 (3.6) | 3.4b(1.2) |
| 6 – 9 | 5.8 (2.0) | 6.3 (2.6) | **10.1 (2.3)** | 15.3 (4.4) | 3.4 (1.3) |
| >=10 | 6.0 (2.2) | 6.2 (2.1) | **9.1 (2.1)** | 16.0 (3.2) | 3.4 (1.1) |
| **Salary range** |  |  |  |  |  |
| <10,000 | 5.5 (1.8) | 6.4 (2.0) | 9.3 (2.3) | 15.6 (3.7) | 3.6 (1.1) |
| 10,000 – 20,000 | 5.9 (2.3) | 6.3 (2.1) | 9.2 (2.2) | 16.2 (3.5) | 3.5 (1.1) |
| >20,000 | 6.6 (1.9) | 5.8 (2.2) | 10.1 (2.1) | 14.6 (3.7) | 3.2 (1.2) |
| **Time from home to PHC centers** | |  |  |  |  |
| 0 – 15 | 6.1 (2.3) | 6.3 (2.4) | 9.0 (2.5) | 15.6 (4.0) | 3.6 (1.1) |
| 16 – 35 | 6.6 (2.2) | 6.1 (2.2) | 9.5 (2.1) | 15.5 (3.8) | 3.4 (1.1) |
| 36 – 55 | 5.3 (1.8) | 5.9 (1.7) | 9.5 (2.3) | 15.9 (2.8) | 3.2 (1.1) |
| >55 | 5.9 (1.9) | 6.9 (1.7) | 9.8 (1.9) | 15.9 (3.8) | 3.7 (1.3) |
| **Shift Time** |  |  |  |  |  |
| Morning | 6.0 (2.0) | 6.2 (2.1) | 9.5 (2.2) | 15.7 (3.7) | 3.4 (1.1) |
| Afternoon | 7.7 (4.0) | 8.3 (1.5) | 6.7(0.6) | 17.0 (4.0) | 4.3 (0.6) |
| Evening | 6.2 (2.6) | 6.2 (2.2) | 8.9 (2.8) | 15.9 (2.8) | 3.3 (1.3) |
| **Working Hour** |  |  |  |  |  |
| 8 hours | 6.0 (2.1) | 6.2 (2.1) | 9.4 (2.3) | 15.7 (3.6) | 3.4 (1.1) |
| 12 hours | 6.5 (5.0) | 6.5 (2.1) | 11.0 (0.00) | 14.5 (5.0) | 3.5 (0.7) |

**Table s2: Study survey questions**

| **Part 1: Job Satisfactions questions To how extend you agree with following statements** | | | | | |
| --- | --- | --- | --- | --- | --- |
| 1.     I feel I am being paid a fair amount for the work I do | Strongly Agree | Agree | Neutral | Disagree | Strongly Disagree |
| 2.     I feel satisfied with my chances for salary increases. | Strongly Agree | Agree | Neutral | Disagree | Strongly Disagree |
| 3.     I feel satisfied with my chances for promotion. | Strongly Agree | Agree | Neutral | Disagree | Strongly Disagree |
| 4.     I feel satisfied with the benefit package I receive. (e.g. health insurance) | Strongly Agree | Agree | Neutral | Disagree | Strongly Disagree |
| 5.     I feel that I have open communication with my top management. | Strongly Agree | Agree | Neutral | Disagree | Strongly Disagree |
| 6.     I feel satisfied with how often I get help and support from management. | Strongly Agree | Agree | Neutral | Disagree | Strongly Disagree |
| 7.     I am regularly informed about important decisions, changes, and plans for the future of the PHCs in SA. | Strongly Agree | Agree | Neutral | Disagree | Strongly Disagree |
| 8.     I feel that the work I do is appreciated. | Strongly Agree | Agree | Neutral | Disagree | Strongly Disagree |
| 9.     I like my direct supervisor. | Strongly Agree | Agree | Neutral | Disagree | Strongly Disagree |
| 10.  I feel my efforts are acknowledged | Strongly Agree | Agree | Neutral | Disagree | Strongly Disagree |
| 11. Policies and procedures in my PHC make me do my job better. | Strongly Agree | Agree | Neutral | Disagree | Strongly Disagree |
| 12.  I feel the goals of PHC are clear to me. | Strongly Agree | Agree | Neutral | Disagree | Strongly Disagree |
| 13.  I feel satisfied with the support I get from my coworkers/team. | Strongly Agree | Agree | Neutral | Disagree | Strongly Disagree |
| 14.  I feel that I have to work harder to overcome the incompetency of my co-workers. | Strongly Agree | Agree | Neutral | Disagree | Strongly Disagree |
| 15.  I feel there is too much bickering and fighting at work. | Strongly Agree | Agree | Neutral | Disagree | Strongly Disagree |
| 16. I feel that sometimes my job is meaningless. | Strongly Agree | Agree | Neutral | Disagree | Strongly Disagree |
| 17.  I feel that I have too much to do at work. | Strongly Agree | Agree | Neutral | Disagree | Strongly Disagree |
| 18.  I feel satisfied with the physical working conditions in my PHC | Strongly Agree | Agree | Neutral | Disagree | Strongly Disagree |
| 19.  Overall I like my work | Strongly Agree | Agree | Neutral | Disagree | Strongly Disagree |
| 20.  I feel that my job description and work tasks are clear to me. | Strongly Agree | Agree | Neutral | Disagree | Strongly Disagree |
| 21.  I feel that I am fully responsible for my work. | Strongly Agree | Agree | Neutral | Disagree | Strongly Disagree |
| 22.  I feel that my role in my PHC has an effect on public health prevention. | Strongly Agree | Agree | Neutral | Disagree | Strongly Disagree |
| 23.  I feel my organization is well-equipped with necessary medical equipment. | Strongly Agree | Agree | Neutral | Disagree | Strongly Disagree |
| 24.  I feel satisfied with the time spent with the patient during the visit | Strongly Agree | Agree | Neutral | Disagree | Strongly Disagree |
| 25.  I feel that the patient and I have the freedom to make his/her care decisions | Strongly Agree | Agree | Neutral | Disagree | Strongly Disagree |
| 26.  Shared-decision making is a priority for me in patient care. | Strongly Agree | Agree | Neutral | Disagree | Strongly Disagree |
| 27.  I feel satisfied with the time I have for my social commitments | Strongly Agree | Agree | Neutral | Disagree | Strongly Disagree |
| 28.  I would recommend employment at PHCs to my colleagues | Strongly Agree | Agree | Neutral | Disagree | Strongly Disagree |
| **29.  Overall, I am satisfied with my employment in PHC.** | Strongly Agree | Agree | Neutral | Disagree | Strongly Disagree |

| **Part 2: General questions** | | | |
| --- | --- | --- | --- |
| **1.     Gender** | Male | Female |  |
| **2.     Age** | 21-30 | 41-50 | > 60 |
|  | 31-40 | 51- 60 |  |
| **3.     Marital status** | Single | Divorce |  |
|  | Married | Widow |  |
| **4.     Specialty** | Physician | Lab Specialist |  |
|  | Nurse | Radiology Specialist |  |
|  | Pharmacist | Others |  |
| **5.     Educational level** | Diploma | Master |  |
|  | Bachelor degree | PhD |  |
| **6.     Salary range** | <10,000 |  |  |
|  | 10,000-20,000 |  |  |
|  | >20,000 |  |  |
| **7.     How long does it take you to arrive to work from your home** | 0-15 minutes | 36-55 minutes |  |
|  | 16-35 minutes | >55 minutes |  |
| **8.     Working shift period** | Morning |  |  |
|  | Afternoon |  |  |
|  | Evening |  |  |
| **9.     Hours of work** | 8 hours |  |  |
|  | 12 hours |  |  |
| **10.  Years of experience** | <= 1 year |  |  |
|  | 2-5 years |  |  |
|  | 6-9 years |  |  |
|  | >= 10 years |  |  |
| **11.  Have you thought of leaving your job during the last two years** | Yes |  |  |
|  | No |  |  |
