# Supplementary material for: The effect of transformation policies on healthcare providers’ satisfaction in primary healthcare centers: the case of Eastern Saudi Arabia
Source: BMC Health Serv Res. 2023 Nov 30;23:1328. doi: 10.1186/s12913-023-10335-8 (PMC10691092; doi:10.1186/s12913-023-10335-8)
Supplement: Supplementary file 2 — Additional file 2: Supplementary 2. Database of Healthcare providers' job satisfaction in primary healthcare centers. [file 12913_2023_10335_MOESM2_ESM.pdf]

Supplementary 2: Database of Healthcare providers' job satisfaction in primary healthcare centers

This file contains individual-level datasets and a summary of agreement levels (ranging from 5 for "strongly agree" to 1 for "strongly disagree") for each question related to the study's main 11 domains. The questions were as follows:

1. I feel I am being paid a fair amount for the work I do
2. I feel satisfied with my chances for salary increases.
3. I feel satisfied with my chances for promotion.
4. I feel satisfied with the benefit package I receive. (e.g. health insurance)
5. I feel that I have open communication with my top management.
6. I feel satisfied with how often I get help and support from management.
7. I am regularly informed about important decisions, changes, and plans for the future of the PHCs in SA.
8. I feel that the work I do is appreciated.
9. I like my direct supervisor.
10. I feel my efforts are acknowledged
11. Policies and procedures in my PHC make me do my job better.
12. I feel the goals of PHC are clear to me.
13. I feel satisfied with the support I get from my coworkers/team.
14. I feel that I have to work harder to overcome the incompetency of my co-workers.
15. I feel there is too much bickering and fighting at work.
16. I feel that sometimes my job is meaningless.
17. I feel that I have too much to do at work.
18. I feel satisfied with the physical working conditions in my PHC
19. Overall I like my work
20. I feel that my job description and work tasks are clear to me.
21. I feel that I am fully responsible for my work.
22. I feel that my role in my PHC has an effect on public health prevention.
23. I feel my organization is well-equipped with necessary medical equipment.
24. I feel satisfied with the time spent with the patient during the visit
25. I feel that the patient and I have the freedom to make his/her care decisions
26. Shared-decision making is a priority for me in patient care.
27. I feel satisfied with the time I have for my social commitments
28. I would recommend employment at PHCs to my colleagues
29. Overall, I am satisfied with my employment in PHC.

ResponseID	Fair_paid	Fair_paid_	Salary_incr	Salary_incr	Chance_pr	Chance_pr	Benifit_ba	Benefit_pa
102334485	3	3	1	5	1	5	1	5
102333836	5	1	3	3	3	3	1	5
102335284	5	1	5	1	5	1	1	5
102335616	1	5	1	5	1	5	1	5
103111695	2	4	2	4	3	3	2	4
103111712	2	4	1	5	4	2	1	5
103608743	4	2	4	2	4	2	2	4
103612209	2	4	2	4	1	5	2	4
103774944	4	2	3	3	3	3	2	4
103774985	2	4	3	3	3	3	3	3
103775094	3	3	3	3	3	3	4	2
103839209	3	3	3	3	2	4	2	4
103840221	3	3	3	3	3	3	2	4
104711072	4	2	4	2	4	2	5	1
105021185	3	3	4	2	4	2	2	4
105022028	2	4	4	2	3	3	4	2
105123427	1	5	2	4	3	3	1	5
105123593	3	3	2	4	2	4	2	4
105021851	2	4	2	4	2	4	2	4
104994850	4	2	4	2	5	1	5	1
102332615	2	4	3	3	3	3	2	4
102332821	2	4	3	3	4	2	2	4
103774198	1	5	4	2	3	3	1	5
103775020	2	4	1	5	2	4	2	4
103775097	5	1	4	2	4	2	4	2
103838903	2	4	2	4	2	4	2	4
103839803	3	3	5	1	5	1	5	1
103840295	5	1	5	1	5	1	1	5
103840379	3	3	5	1	5	1	3	3
103854842	3	3	4	2	4	2	2	4
104611172	2	4	3	3	3	3	2	4
104986449	2	4	3	3	2	4	2	4
104991362	4	2	4	2	4	2	3	3
104994284	3	3	3	3	2	4	2	4
105020222	4	2	5	1	5	1	3	3
105020383	2	4	4	2	4	2	1	5
105021126	2	4	3	3	3	3	2	4
105021177	5	1	4	2	2	4	3	3
105021281	2	4	2	4	2	4	2	4
105021334	2	4	1	5	1	5	1	5
105022210	2	4	2	4	2	4	2	4
105031981	2	4	2	4	3	3	1	5
105184089	2	4	2	4	2	4	2	4
105249126	2	4	3	3	4	2	2	4
105251530	5	1	4	2	3	3	4	2
105252492	2	4	5	1	3	3	2	4
105253385	4	2	3	3	3	3	3	3
105258597	5	1	2	4	5	1	1	5
102332214	2	4	3	3	2	4	2	4

102335694	3	3	4	2	4	2	3	3
104984903	2	4	3	3	3	3	2	4
105023734	2	4	3	3	4	2	2	4
105123302	2	4	2	4	2	4	2	4
105123774	3	3	3	3	4	2	1	5
105152699	4	2	4	2	4	2	2	4
105250107	4	2	5	1	4	2	5	1
105251014	5	1	4	2	4	2	4	2
104985420	3	3	1	5	1	5	1	5
102334856	1	5	1	5	1	5	1	5
103111795	2	4	2	4	3	3	2	4
102422528	5	1	5	1	5	1	4	2
103773475	3	3	3	3	1	5	3	3
105020842	4	2	2	4	2	4	2	4
105021246	4	2	5	1	3	3	5	1
105021926	2	4	3	3	5	1	5	1
105021984	3	3	3	3	3	3	3	3
105022119	3	3	3	3	3	3	3	3
105026687	2	4	2	4	2	4	2	4
105021556	3	3	2	4	3	3	4	2
102480197	3	3	3	3	3	3	4	2
102788147	4	2	2	4	1	5	1	5
103773458	3	3	3	3	3	3	3	3
103774017	4	2	3	3	2	4	2	4
103774065	2	4	3	3	3	3	3	3
103775026	3	3	3	3	2	4	3	3
104987072	3	3	2	4	4	2	5	1
104987637	5	1	5	1	2	4	5	1
104989206	3	3	4	2	2	4	5	1
104989207	4	2	2	4	1	5	1	5
105019523	4	2	2	4	2	4	4	2
105020094	4	2	4	2	4	2	5	1
105020140	2	4	1	5	1	5	1	5
105020202	2	4	1	5	2	4	4	2
105020308	3	3	2	4	2	4	2	4
105020721	3	3	1	5	1	5	1	5
105021504	2	4	4	2	4	2	5	1
105021869	2	4	2	4	2	4	4	2
105022078	3	3	2	4	3	3	3	3
105022183	4	2	3	3	2	4	5	1
105025427	3	3	2	4	2	4	5	1
105025560	2	4	2	4	2	4	5	1
105030621	2	4	2	4	2	4	5	1
105123819	4	2	4	2	2	4	1	5
105153197	4	2	2	4	2	4	5	1
105214326	3	3	1	5	1	5	5	1
105214457	1	5	1	5	1	5	1	5
105249133	5	1	5	1	4	2	5	1
105249298	2	4	3	3	3	3	5	1
105250395	2	4	2	4	3	3	4	2

105251194	4	2	4	2	4	2	4	2
105251516	4	2	4	2	4	2	5	1
105251882	2	4	2	4	4	2	5	1
105252047	2	4	4	2	4	2	4	2
105024326	2	4	2	4	2	4	5	1
103773075	2	4	2	4	4	2	5	1
102346665	2	4	2	4	2	4	2	4
103774016	3	3	3	3	3	3	3	3
104985652	4	2	4	2	4	2	5	1
104985812	2	4	2	4	2	4	5	1
104988625	4	2	2	4	2	4	2	4
105019417	4	2	1	5	1	5	4	2
105020524	3	3	4	2	4	2	4	2
105127068	2	4	2	4	2	4	5	1
105131564	3	3	2	4	2	4	5	1
102358824	3	3	2	4	2	4	2	4
102357992	1	5	1	5	1	5	1	5
104987473	4	2	2	4	2	4	4	2
102378993	2	4	2	4	3	3	4	2
102379239	3	3	2	4	4	2	4	2
105122262	4	2	4	2	4	2	5	1
105263379	2	4	3	3	3	3	5	1
103629908	5	1	4	2	4	2	2	4
105214826	2	4	2	4	4	2	4	2
103608770	4	2	1	5	3	3	4	2
104985561	2	4	1	5	1	5	1	5
103773703	3	3	3	3	3	3	1	5
103838737	3	3	4	2	3	3	3	3
105252197	2	4	2	4	4	2	4	2
105214959	3	3	3	3	3	3	3	3
104985127	2	4	2	4	2	4	3	3
105249467	5	1	5	1	5	1	5	1
105214475	5	1	4	2	5	1	5	1
105214991	2	4	4	2	4	2	4	2
105249130	4	2	2	4	2	4	1	5
105251665	2	4	4	2	4	2	5	1
105561007	4	2	4	2	4	2	4	2
105249163	3	3	3	3	4	2	3	3
105249960	2	4	2	4	2	4	3	3
105253865	3	3	3	3	4	2	4	2
105253658	4	2	4	2	4	2	4	2
105412749	4	2	5	1	4	2	5	1
103774515	2	4	1	5	4	2	3	3
105024472	2	4	4	2	4	2	5	1

Communic	Communic	Help_supp	Help_supp	Informed_	Informed_	Work_app	Work_app	Direct_sup
2	4	2	4	3	3	2	4	2
1	5	2	4	2	4	2	4	1
4	2	4	2	2	4	5	1	5
3	3	2	4	1	5	1	5	1
3	3	3	3	4	2	3	3	2
4	2	3	3	5	1	4	2	2
2	4	2	4	5	1	3	3	1
2	4	1	5	1	5	2	4	2
2	4	2	4	2	4	2	4	2
2	4	2	4	2	4	2	4	2
1	5	1	5	3	3	2	4	1
2	4	2	4	3	3	3	3	3
4	2	2	4	4	2	2	4	3
4	2	3	3	4	2	3	3	4
3	3	3	3	4	2	2	4	3
2	4	3	3	3	3	3	3	2
4	2	3	3	3	3	3	3	2
2	4	3	3	3	3	2	4	2
3	3	3	3	4	2	4	2	2
5	1	5	1	4	2	5	1	5
3	3	2	4	3	3	3	3	2
3	3	4	2	2	4	4	2	2
3	3	2	4	4	2	2	4	1
2	4	2	4	2	4	2	4	2
4	2	4	2	3	3	3	3	4
2	4	3	3	3	3	3	3	1
5	1	5	1	3	3	4	2	2
5	1	5	1	3	3	3	3	5
3	3	3	3	4	2	3	3	1
3	3	3	3	4	2	4	2	2
1	5	2	4	2	4	2	4	1
3	3	4	2	4	2	4	2	2
5	1	4	2	4	2	5	1	4
2	4	4	2	4	2	2	4	3
4	2	2	4	2	4	3	3	1
2	4	2	4	4	2	3	3	2
3	3	3	3	4	2	3	3	1
2	4	2	4	2	4	4	2	1
1	5	1	5	2	4	1	5	1
2	4	1	5	2	4	2	4	1
2	4	2	4	1	5	1	5	1
3	3	3	3	3	3	1	5	2
2	4	2	4	2	4	2	4	2
3	3	2	4	2	4	2	4	2
4	2	4	2	4	2	2	4	4
5	1	5	1	4	2	5	1	1
5	1	5	1	3	3	5	1	2
4	2	2	4	2	4	3	3	1
5	1	5	1	2	4	2	4	4

5	1	5	1	5	1	5	1	5
2	4	2	4	2	4	2	4	1
3	3	3	3	2	4	4	2	4
3	3	3	3	5	1	3	3	3
2	4	2	4	2	4	2	4	2
4	2	4	2	4	2	4	2	4
3	3	3	3	4	2	3	3	3
3	3	3	3	4	2	2	4	2
2	4	3	3	1	5	2	4	1
1	5	1	5	1	5	1	5	1
3	3	3	3	4	2	4	2	2
5	1	5	1	1	5	4	2	5
1	5	1	5	1	5	3	3	1
3	3	3	3	3	3	5	1	5
3	3	2	4	2	4	3	3	2
1	5	3	3	2	4	2	4	1
3	3	3	3	3	3	3	3	3
2	4	2	4	3	3	4	2	2
2	4	2	4	2	4	2	4	2
2	4	2	4	3	3	3	3	2
2	4	2	4	2	4	2	4	1
3	3	2	4	2	4	2	4	2
3	3	3	3	3	3	3	3	1
2	4	2	4	2	4	2	4	2
3	3	4	2	2	4	1	5	5
3	3	3	3	2	4	3	3	2
5	1	5	1	4	2	4	2	4
4	2	3	3	4	2	3	3	2
2	4	2	4	4	2	2	4	1
3	3	3	3	1	5	3	3	1
2	4	4	2	5	1	4	2	2
4	2	2	4	2	4	2	4	1
2	4	1	5	1	5	2	4	1
2	4	4	2	2	4	2	4	2
3	3	2	4	2	4	3	3	2
2	4	2	4	3	3	3	3	1
4	2	4	2	2	4	4	2	2
3	3	3	3	3	3	4	2	2
3	3	3	3	3	3	4	2	2
5	1	5	1	3	3	3	3	5
4	2	4	2	4	2	2	4	3
2	4	3	3	3	3	3	3	2
4	2	3	3	3	3	3	3	2
3	3	3	3	4	2	4	2	3
2	4	2	4	2	4	2	4	2
5	1	4	2	5	1	5	1	1
3	3	3	3	3	3	2	4	4
4	2	4	2	4	2	4	2	2
4	2	4	2	4	2	5	1	3
5	1	5	1	2	4	5	1	3

4	2	3	3	3	3	3	3	1
3	3	2	4	3	3	3	3	2
4	2	4	2	4	2	4	2	4
4	2	4	2	4	2	2	4	2
3	3	2	4	4	2	1	5	1
2	4	2	4	2	4	2	4	2
2	4	2	4	3	3	3	3	1
3	3	3	3	3	3	3	3	1
3	3	3	3	3	3	3	3	1
5	1	3	3	3	3	3	3	4
2	4	2	4	2	4	2	4	2
1	5	1	5	1	5	2	4	2
3	3	2	4	4	2	2	4	2
4	2	4	2	2	4	3	3	1
4	2	3	3	3	3	2	4	1
3	3	3	3	4	2	3	3	3
1	5	1	5	1	5	1	5	1
2	4	2	4	5	1	2	4	1
5	1	4	2	4	2	5	1	5
4	2	4	2	3	3	3	3	3
5	1	5	1	5	1	5	1	3
3	3	2	4	4	2	3	3	3
3	3	3	3	3	3	4	2	2
3	3	2	4	4	2	2	4	1
2	4	1	5	1	5	1	5	1
3	3	3	3	2	4	4	2	4
3	3	3	3	3	3	3	3	1
3	3	3	3	2	4	3	3	2
2	4	5	1	5	1	5	1	2
2	4	2	4	2	4	2	4	2
1	5	1	5	2	4	1	5	1
5	1	5	1	5	1	5	1	3
2	4	2	4	4	2	2	4	1
1	5	2	4	3	3	2	4	2
4	2	2	4	2	4	1	5	1
4	2	4	2	4	2	4	2	2
4	2	4	2	4	2	4	2	4
3	3	1	5	2	4	2	4	1
3	3	2	4	1	5	2	4	2
4	2	5	1	4	2	5	1	3
5	1	5	1	2	4	4	2	3
5	1	5	1	5	1	2	4	2
2	4	1	5	1	5	2	4	2
4	2	3	3	4	2	5	1	3

Direct_sup	Effort_ackr	Effort_ackr	Policies_pr	Policies_pr	Goal_clear	Goal_clear	Support_co	Support_co
4	2	4	2	4	2	4	2	4
5	3	3	3	3	3	3	2	4
1	4	2	5	1	3	3	1	5
5	1	5	1	5	1	5	1	5
4	2	4	2	4	2	4	1	5
4	4	2	1	5	4	2	3	3
5	2	4	4	2	4	2	3	3
4	2	4	3	3	3	3	2	4
4	2	4	2	4	2	4	2	4
4	2	4	3	3	3	3	2	4
5	2	4	3	3	3	3	1	5
3	2	4	3	3	1	5	1	5
3	3	3	4	2	4	2	2	4
2	5	1	4	2	4	2	2	4
3	2	4	2	4	3	3	2	4
4	2	4	2	4	2	4	2	4
4	3	3	3	3	3	3	4	2
4	2	4	2	4	2	4	3	3
4	3	3	3	3	3	3	2	4
1	5	1	5	1	5	1	5	1
4	2	4	2	4	2	4	2	4
4	2	4	4	2	4	2	2	4
5	2	4	4	2	1	5	1	5
4	3	3	3	3	3	3	3	3
2	3	3	4	2	5	1	3	3
5	2	4	3	3	3	3	2	4
4	3	3	5	1	3	3	2	4
1	5	1	5	1	5	1	1	5
5	3	3	4	2	2	4	2	4
4	3	3	3	3	3	3	3	3
5	2	4	2	4	2	4	2	4
4	3	3	2	4	2	4	2	4
2	4	2	4	2	4	2	3	3
3	4	2	5	1	4	2	2	4
5	3	3	2	4	3	3	1	5
4	4	2	3	3	3	3	2	4
5	3	3	4	2	4	2	2	4
5	2	4	5	1	3	3	1	5
5	1	5	1	5	1	5	1	5
5	2	4	3	3	4	2	1	5
5	2	4	1	5	1	5	1	5
4	1	5	4	2	2	4	1	5
4	2	4	2	4	2	4	2	4
4	3	3	3	3	3	3	2	4
2	4	2	4	2	4	2	2	4
5	5	1	5	1	3	3	1	5
4	5	1	5	1	4	2	3	3
5	4	2	5	1	4	2	4	2
2	4	2	4	2	4	2	2	4

1	5	1	5	1	5	1	4	2
5	2	4	2	4	2	4	2	4
2	4	2	4	2	2	4	3	3
3	3	3	4	2	4	2	2	4
4	2	4	1	5	2	4	2	4
2	4	2	3	3	4	2	4	2
3	4	2	4	2	4	2	2	4
4	3	3	3	3	4	2	2	4
5	2	4	2	4	1	5	1	5
5	1	5	1	5	1	5	1	5
4	3	3	3	3	4	2	2	4
1	5	1	5	1	2	4	2	4
5	1	5	1	5	3	3	1	5
1	3	3	3	3	3	3	4	2
4	4	2	2	4	3	3	3	3
5	2	4	2	4	4	2	2	4
3	3	3	3	3	3	3	3	3
4	3	3	3	3	2	4	2	4
4	2	4	2	4	2	4	2	4
4	3	3	3	3	2	4	2	4
5	1	5	1	5	1	5	1	5
4	1	5	3	3	2	4	3	3
5	3	3	3	3	3	3	1	5
4	2	4	2	4	2	4	2	4
1	2	4	5	1	3	3	4	2
4	1	5	1	5	3	3	1	5
2	4	2	2	4	3	3	5	1
4	2	4	3	3	3	3	1	5
5	2	4	2	4	2	4	2	4
5	3	3	3	3	3	3	2	4
4	5	1	4	2	2	4	2	4
5	2	4	4	2	2	4	2	4
5	1	5	1	5	1	5	1	5
4	3	3	4	2	4	2	2	4
4	3	3	2	4	2	4	2	4
5	4	2	3	3	2	4	2	4
4	3	3	4	2	3	3	3	3
4	3	3	3	3	4	2	4	2
4	3	3	3	3	3	3	3	3
1	4	2	4	2	3	3	3	3
3	4	2	4	2	4	2	3	3
4	2	4	3	3	2	4	3	3
4	4	2	2	4	2	4	3	3
3	4	2	2	4	2	4	2	4
4	2	4	2	4	2	4	2	4
5	3	3	4	2	4	2	4	2
2	1	5	3	3	3	3	3	3
4	3	3	5	1	4	2	3	3
3	3	3	5	1	4	2	3	3
3	5	1	4	2	4	2	4	2

5	3	3	2	4	2	4	3	3
4	2	4	4	2	4	2	1	5
2	4	2	4	2	2	4	4	2
4	3	3	3	3	4	2	4	2
5	2	4	2	4	1	5	3	3
4	2	4	3	3	2	4	2	4
5	3	3	3	3	2	4	1	5
5	1	5	3	3	3	3	3	3
5	3	3	3	3	3	3	2	4
2	3	3	4	2	4	2	3	3
4	2	4	2	4	2	4	2	4
4	3	3	1	5	1	5	1	5
4	2	4	2	4	2	4	2	4
5	4	2	2	4	3	3	4	2
5	3	3	2	4	2	4	3	3
3	4	2	4	2	4	2	2	4
5	1	5	1	5	1	5	1	5
5	2	4	3	3	2	4	2	4
1	5	1	4	2	5	1	3	3
3	2	4	3	3	4	2	2	4
3	5	1	2	4	2	4	3	3
3	4	2	4	2	4	2	3	3
4	4	2	2	4	2	4	1	5
5	2	4	2	4	3	3	2	4
5	2	4	1	5	1	5	1	5
2	4	2	2	4	2	4	3	3
5	2	4	3	3	1	5	2	4
4	3	3	3	3	3	3	2	4
4	5	1	3	3	4	2	2	4
4	2	4	2	4	2	4	1	5
5	1	5	1	5	1	5	1	5
3	5	1	5	1	2	4	5	1
5	2	4	3	3	2	4	1	5
4	2	4	2	4	2	4	1	5
5	1	5	1	5	1	5	1	5
4	4	2	5	1	4	2	4	2
2	4	2	4	2	4	2	4	2
5	1	5	1	5	2	4	2	4
4	2	4	2	4	3	3	2	4
3	5	1	4	2	3	3	2	4
3	4	2	5	1	4	2	5	1
4	4	2	5	1	5	1	2	4
4	2	4	2	4	1	5	1	5
3	5	1	5	1	5	1	4	2

Overcome_	Overcome_	Conflict_fi	Conflict_fi	Job_meani	Job_meani	Work_toor	Work_toor	Physical_c
3	3.00	4	4.00	4	4.00	3	3.00	3
1	1.00	5	5.00	5	5.00	2	2.00	2
5	5.00	5	5.00	1	1.00	1	1.00	4
3	3.00	4	4.00	5	5.00	1	1.00	3
3	3.00	1	1.00	1	1.00	2	2.00	4
4	4.00	2	2.00	5	5.00	1	1.00	2
4	4.00	4	4.00	4	4.00	4	4.00	1
4	4.00	3	3.00	4	4.00	2	2.00	4
3	3.00	4	4.00	4	4.00	2	2.00	3
4	4.00	4	4.00	5	5.00	4	4.00	4
4	4.00	5	5.00	5	5.00	4	4.00	2
4	4.00	4	4.00	4	4.00	2	2.00	2
1	1.00	2	2.00	4	4.00	2	2.00	5
2	2.00	5	5.00	5	5.00	3	3.00	5
4	4.00	2	2.00	4	4.00	2	2.00	4
2	2.00	4	4.00	4	4.00	3	3.00	4
3	3.00	2	2.00	5	5.00	1	1.00	4
3	3.00	2	2.00	2	2.00	2	2.00	2
2	2.00	3	3.00	3	3.00	1	1.00	3
1	1.00	1	1.00	2	2.00	1	1.00	4
2	2.00	2	2.00	2	2.00	2	2.00	2
2	2.00	4	4.00	4	4.00	1	1.00	4
3	3.00	4	4.00	2	2.00	2	2.00	4
3	3.00	3	3.00	2	2.00	3	3.00	3
3	3.00	4	4.00	4	4.00	2	2.00	1
3	3.00	3	3.00	3	3.00	2	2.00	4
1	1.00	3	3.00	1	1.00	1	1.00	2
5	5.00	3	3.00	5	5.00	1	1.00	5
3	3.00	4	4.00	3	3.00	1	1.00	4
3	3.00	2	2.00	4	4.00	2	2.00	4
3	3.00	3	3.00	4	4.00	3	3.00	3
2	2.00	3	3.00	4	4.00	1	1.00	4
2	2.00	3	3.00	1	1.00	1	1.00	5
4	4.00	2	2.00	4	4.00	1	1.00	5
5	5.00	4	4.00	5	5.00	1	1.00	3
3	3.00	4	4.00	3	3.00	3	3.00	2
3	3.00	3	3.00	2	2.00	2	2.00	5
1	1.00	3	3.00	1	1.00	3	3.00	5
3	3.00	5	5.00	5	5.00	2	2.00	2
2	2.00	5	5.00	5	5.00	2	2.00	4
1	1.00	1	1.00	1	1.00	1	1.00	1
5	5.00	1	1.00	5	5.00	2	2.00	4
2	2.00	4	4.00	4	4.00	2	2.00	4
5	5.00	4	4.00	5	5.00	3	3.00	3
1	1.00	3	3.00	4	4.00	2	2.00	5
3	3.00	5	5.00	5	5.00	1	1.00	5
4	4.00	2	2.00	2	2.00	2	2.00	5
3	3.00	4	4.00	4	4.00	1	1.00	5
3	3.00	3	3.00	4	4.00	1	1.00	4

2	2.00	1	1.00	1	1.00	1	1.00	5
2	2.00	5	5.00	5	5.00	1	1.00	2
3	3.00	2	2.00	3	3.00	3	3.00	4
3	3.00	4	4.00	3	3.00	2	2.00	4
3	3.00	4	4.00	5	5.00	2	2.00	2
3	3.00	2	2.00	4	4.00	2	2.00	2
3	3.00	3	3.00	3	3.00	3	3.00	2
3	3.00	4	4.00	4	4.00	2	2.00	3
1	1.00	3	3.00	4	4.00	2	2.00	2
2	2.00	5	5.00	5	5.00	1	1.00	1
2	2.00	3	3.00	4	4.00	3	3.00	3
4	4.00	3	3.00	1	1.00	3	3.00	1
3	3.00	1	1.00	1	1.00	3	3.00	1
2	2.00	1	1.00	4	4.00	3	3.00	4
3	3.00	3	3.00	4	4.00	5	5.00	3
3	3.00	2	2.00	5	5.00	4	4.00	2
3	3.00	3	3.00	3	3.00	3	3.00	3
3	3.00	3	3.00	3	3.00	1	1.00	2
2	2.00	4	4.00	4	4.00	3	3.00	3
3	3.00	4	4.00	4	4.00	2	2.00	3
3	3.00	5	5.00	4	4.00	2	2.00	3
2	2.00	1	1.00	4	4.00	2	2.00	3
3	3.00	3	3.00	3	3.00	3	3.00	3
4	4.00	5	5.00	5	5.00	3	3.00	2
4	4.00	2	2.00	5	5.00	1	1.00	4
4	4.00	1	1.00	3	3.00	3	3.00	2
1	1.00	2	2.00	2	2.00	4	4.00	5
4	4.00	4	4.00	4	4.00	3	3.00	5
1	1.00	3	3.00	2	2.00	2	2.00	3
1	1.00	2	2.00	3	3.00	1	1.00	3
2	2.00	2	2.00	2	2.00	1	1.00	4
2	2.00	1	1.00	1	1.00	2	2.00	2
3	3.00	4	4.00	5	5.00	2	2.00	1
2	2.00	2	2.00	4	4.00	2	2.00	4
2	2.00	2	2.00	5	5.00	2	2.00	2
2	2.00	4	4.00	5	5.00	3	3.00	2
3	3.00	2	2.00	3	3.00	1	1.00	5
3	3.00	4	4.00	3	3.00	3	3.00	3
3	3.00	3	3.00	3	3.00	1	1.00	3
1	1.00	1	1.00	4	4.00	1	1.00	4
2	2.00	2	2.00	4	4.00	1	1.00	5
3	3.00	3	3.00	5	5.00	1	1.00	3
4	4.00	2	2.00	4	4.00	2	2.00	2
2	2.00	2	2.00	2	2.00	2	2.00	3
4	4.00	4	4.00	5	5.00	2	2.00	2
3	3.00	4	4.00	4	4.00	4	4.00	5
3	3.00	3	3.00	3	3.00	3	3.00	1
1	1.00	2	2.00	2	2.00	1	1.00	5
1	1.00	1	1.00	4	4.00	1	1.00	4
3	3.00	2	2.00	4	4.00	3	3.00	5

2	2.00	3	3.00	4	4.00	3	3.00	3
2	2.00	3	3.00	4	4.00	2	2.00	5
1	1.00	1	1.00	4	4.00	2	2.00	4
3	3.00	4	4.00	2	2.00	3	3.00	3
1	1.00	2	2.00	5	5.00	2	2.00	2
2	2.00	4	4.00	4	4.00	2	2.00	4
4	4.00	2	2.00	3	3.00	2	2.00	2
3	3.00	1	1.00	3	3.00	3	3.00	3
3	3.00	3	3.00	4	4.00	3	3.00	3
3	3.00	2	2.00	5	5.00	2	2.00	4
2	2.00	4	4.00	4	4.00	4	4.00	2
1	1.00	5	5.00	5	5.00	2	2.00	2
3	3.00	4	4.00	4	4.00	2	2.00	4
4	4.00	4	4.00	5	5.00	2	2.00	2
2	2.00	4	4.00	5	5.00	2	2.00	3
2	2.00	3	3.00	4	4.00	2	2.00	3
1	1.00	1	1.00	4	4.00	2	2.00	1
2	2.00	4	4.00	4	4.00	2	2.00	4
2	2.00	2	2.00	2	2.00	1	1.00	4
3	3.00	4	4.00	4	4.00	3	3.00	3
2	2.00	2	2.00	2	2.00	2	2.00	5
3	3.00	4	4.00	3	3.00	2	2.00	2
3	3.00	5	5.00	3	3.00	1	1.00	2
4	4.00	4	4.00	4	4.00	3	3.00	2
1	1.00	4	4.00	4	4.00	3	3.00	1
3	3.00	2	2.00	2	2.00	2	2.00	2
3	3.00	3	3.00	3	3.00	2	2.00	3
2	2.00	5	5.00	3	3.00	3	3.00	2
3	3.00	2	2.00	2	2.00	3	3.00	5
1	1.00	4	4.00	4	4.00	3	3.00	2
2	2.00	4	4.00	4	4.00	1	1.00	2
1	1.00	5	5.00	1	1.00	3	3.00	5
4	4.00	4	4.00	4	4.00	2	2.00	5
4	4.00	4	4.00	5	5.00	3	3.00	2
1	1.00	1	1.00	4	4.00	1	1.00	5
2	2.00	2	2.00	2	2.00	3	3.00	4
3	3.00	3	3.00	3	3.00	3	3.00	4
2	2.00	3	3.00	4	4.00	3	3.00	2
3	3.00	4	4.00	5	5.00	2	2.00	3
4	4.00	2	2.00	1	1.00	3	3.00	5
3	3.00	3	3.00	2	2.00	3	3.00	5
1	1.00	1	1.00	1	1.00	1	1.00	5
3	3.00	3	3.00	5	5.00	4	4.00	3
2	2.00	2	2.00	4	4.00	2	2.00	5

Physical_c	Like_work	Like_work_	Job_descri	Job_descri	Responsibl	Responsibl	Public_hea	Public_hea
3.00	2	4.00	3	3.00	2	4.00	4	2.00
4.00	2	4.00	1	5.00	1	5.00	1	5.00
2.00	5	1.00	2	4.00	1	5.00	1	5.00
3.00	1	5.00	1	5.00	1	5.00	1	5.00
2.00	1	5.00	2	4.00	2	4.00	2	4.00
4.00	1	5.00	2	4.00	1	5.00	1	5.00
5.00	2	4.00	2	4.00	2	4.00	2	4.00
2.00	2	4.00	2	4.00	1	5.00	3	3.00
3.00	2	4.00	2	4.00	2	4.00	2	4.00
2.00	2	4.00	2	4.00	2	4.00	2	4.00
4.00	2	4.00	2	4.00	1	5.00	1	5.00
4.00	2	4.00	2	4.00	2	4.00	2	4.00
1.00	2	4.00	4	2.00	2	4.00	2	4.00
1.00	3	3.00	3	3.00	2	4.00	4	2.00
2.00	2	4.00	4	2.00	2	4.00	2	4.00
2.00	2	4.00	2	4.00	2	4.00	2	4.00
2.00	4	2.00	1	5.00	1	5.00	1	5.00
4.00	1	5.00	3	3.00	3	3.00	2	4.00
3.00	3	3.00	2	4.00	2	4.00	1	5.00
2.00	5	1.00	3	3.00	2	4.00	2	4.00
4.00	2	4.00	3	3.00	2	4.00	2	4.00
2.00	2	4.00	2	4.00	2	4.00	2	4.00
2.00	1	5.00	1	5.00	1	5.00	2	4.00
3.00	2	4.00	3	3.00	3	3.00	3	3.00
5.00	3	3.00	4	2.00	5	1.00	2	4.00
2.00	3	3.00	3	3.00	3	3.00	2	4.00
4.00	3	3.00	5	1.00	2	4.00	3	3.00
1.00	2	4.00	5	1.00	5	1.00	1	5.00
2.00	1	5.00	4	2.00	2	4.00	1	5.00
2.00	3	3.00	5	1.00	2	4.00	2	4.00
3.00	2	4.00	2	4.00	3	3.00	2	4.00
2.00	2	4.00	4	2.00	2	4.00	2	4.00
1.00	5	1.00	4	2.00	3	3.00	3	3.00
1.00	4	2.00	4	2.00	2	4.00	3	3.00
3.00	2	4.00	5	1.00	1	5.00	2	4.00
4.00	4	2.00	2	4.00	2	4.00	2	4.00
1.00	3	3.00	3	3.00	3	3.00	2	4.00
1.00	2	4.00	1	5.00	2	4.00	3	3.00
4.00	1	5.00	1	5.00	1	5.00	1	5.00
2.00	1	5.00	2	4.00	3	3.00	1	5.00
5.00	1	5.00	1	5.00	1	5.00	1	5.00
2.00	2	4.00	3	3.00	1	5.00	2	4.00
2.00	1	5.00	3	3.00	2	4.00	1	5.00
3.00	2	4.00	2	4.00	1	5.00	1	5.00
1.00	3	3.00	4	2.00	2	4.00	2	4.00
1.00	3	3.00	2	4.00	1	5.00	1	5.00
1.00	4	2.00	2	4.00	2	4.00	1	5.00
1.00	1	5.00	5	1.00	1	5.00	1	5.00
2.00	3	3.00	4	2.00	3	3.00	2	4.00

1.00	4	2.00	5	1.00	2	4.00	1	5.00
4.00	1	5.00	2	4.00	1	5.00	1	5.00
2.00	2	4.00	3	3.00	3	3.00	2	4.00
2.00	3	3.00	3	3.00	3	3.00	2	4.00
4.00	1	5.00	1	5.00	1	5.00	1	5.00
4.00	2	4.00	3	3.00	2	4.00	2	4.00
4.00	1	5.00	3	3.00	3	3.00	1	5.00
3.00	2	4.00	2	4.00	3	3.00	2	4.00
4.00	1	5.00	1	5.00	1	5.00	1	5.00
5.00	1	5.00	1	5.00	1	5.00	1	5.00
3.00	3	3.00	4	2.00	2	4.00	2	4.00
5.00	5	1.00	2	4.00	2	4.00	5	1.00
5.00	1	5.00	1	5.00	1	5.00	3	3.00
2.00	3	3.00	4	2.00	2	4.00	2	4.00
3.00	1	5.00	3	3.00	1	5.00	2	4.00
4.00	2	4.00	2	4.00	2	4.00	1	5.00
3.00	3	3.00	3	3.00	3	3.00	3	3.00
4.00	2	4.00	2	4.00	1	5.00	2	4.00
3.00	2	4.00	2	4.00	2	4.00	2	4.00
3.00	2	4.00	2	4.00	2	4.00	3	3.00
3.00	2	4.00	2	4.00	2	4.00	2	4.00
3.00	1	5.00	2	4.00	2	4.00	3	3.00
3.00	1	5.00	3	3.00	3	3.00	3	3.00
4.00	2	4.00	2	4.00	1	5.00	2	4.00
2.00	3	3.00	3	3.00	1	5.00	1	5.00
4.00	2	4.00	2	4.00	1	5.00	3	3.00
1.00	3	3.00	2	4.00	2	4.00	2	4.00
1.00	2	4.00	2	4.00	2	4.00	2	4.00
3.00	2	4.00	2	4.00	2	4.00	2	4.00
3.00	2	4.00	3	3.00	2	4.00	3	3.00
2.00	4	2.00	2	4.00	2	4.00	2	4.00
4.00	1	5.00	1	5.00	1	5.00	2	4.00
5.00	1	5.00	1	5.00	1	5.00	1	5.00
2.00	1	5.00	3	3.00	1	5.00	2	4.00
4.00	2	4.00	2	4.00	2	4.00	2	4.00
4.00	1	5.00	2	4.00	2	4.00	2	4.00
1.00	2	4.00	2	4.00	2	4.00	2	4.00
3.00	3	3.00	2	4.00	2	4.00	3	3.00
3.00	3	3.00	3	3.00	3	3.00	3	3.00
2.00	3	3.00	5	1.00	1	5.00	1	5.00
1.00	2	4.00	3	3.00	2	4.00	1	5.00
3.00	1	5.00	2	4.00	2	4.00	2	4.00
4.00	2	4.00	2	4.00	2	4.00	2	4.00
3.00	4	2.00	2	4.00	2	4.00	4	2.00
4.00	1	5.00	2	4.00	2	4.00	2	4.00
1.00	5	1.00	5	1.00	1	5.00	4	2.00
5.00	2	4.00	4	2.00	1	5.00	3	3.00
1.00	2	4.00	4	2.00	2	4.00	2	4.00
2.00	1	5.00	4	2.00	1	5.00	2	4.00
1.00	4	2.00	4	2.00	3	3.00	3	3.00

3.00	2	4.00	2	4.00	2	4.00	2	4.00
1.00	3	3.00	2	4.00	2	4.00	2	4.00
2.00	2	4.00	1	5.00	1	5.00	1	5.00
3.00	2	4.00	5	1.00	5	1.00	2	4.00
4.00	1	5.00	1	5.00	1	5.00	1	5.00
2.00	2	4.00	2	4.00	2	4.00	2	4.00
4.00	2	4.00	3	3.00	2	4.00	3	3.00
3.00	3	3.00	3	3.00	3	3.00	2	4.00
3.00	2	4.00	2	4.00	2	4.00	3	3.00
2.00	2	4.00	2	4.00	2	4.00	2	4.00
4.00	2	4.00	2	4.00	2	4.00	2	4.00
4.00	1	5.00	1	5.00	1	5.00	1	5.00
2.00	1	5.00	2	4.00	2	4.00	2	4.00
4.00	1	5.00	2	4.00	2	4.00	3	3.00
3.00	2	4.00	3	3.00	2	4.00	1	5.00
3.00	1	5.00	4	2.00	2	4.00	2	4.00
5.00	1	5.00	1	5.00	1	5.00	1	5.00
2.00	2	4.00	2	4.00	2	4.00	2	4.00
2.00	4	2.00	4	2.00	2	4.00	3	3.00
3.00	4	2.00	3	3.00	2	4.00	2	4.00
1.00	1	5.00	2	4.00	2	4.00	3	3.00
4.00	2	4.00	2	4.00	2	4.00	2	4.00
4.00	3	3.00	1	5.00	1	5.00	1	5.00
4.00	2	4.00	2	4.00	2	4.00	3	3.00
5.00	2	4.00	1	5.00	1	5.00	1	5.00
4.00	2	4.00	2	4.00	2	4.00	2	4.00
3.00	3	3.00	2	4.00	2	4.00	3	3.00
4.00	3	3.00	3	3.00	3	3.00	3	3.00
1.00	2	4.00	4	2.00	1	5.00	2	4.00
4.00	3	3.00	2	4.00	2	4.00	2	4.00
4.00	1	5.00	2	4.00	2	4.00	3	3.00
1.00	3	3.00	5	1.00	4	2.00	3	3.00
1.00	2	4.00	2	4.00	2	4.00	2	4.00
4.00	2	4.00	2	4.00	2	4.00	1	5.00
1.00	1	5.00	1	5.00	1	5.00	1	5.00
2.00	2	4.00	2	4.00	3	3.00	3	3.00
2.00	3	3.00	3	3.00	3	3.00	3	3.00
4.00	2	4.00	2	4.00	2	4.00	3	3.00
3.00	2	4.00	2	4.00	2	4.00	2	4.00
1.00	4	2.00	4	2.00	4	2.00	3	3.00
1.00	3	3.00	4	2.00	3	3.00	3	3.00
1.00	5	1.00	5	1.00	2	4.00	2	4.00
3.00	1	5.00	1	5.00	1	5.00	4	2.00
1.00	3	3.00	3	3.00	2	4.00	1	5.00

Medical_e	Medicl_eq	Patient_tir	Patient_tir	Freedom_c	Freedom_c	Shared_de	Shared_de	Social_com
2	4.00	2	4.00	2	4.00	2	4.00	1
3	3.00	2	4.00	2	4.00	2	4.00	3
4	2.00	5	1.00	1	5.00	1	5.00	5
2	4.00	1	5.00	1	5.00	1	5.00	1
3	3.00	3	3.00	2	4.00	2	4.00	4
3	3.00	3	3.00	4	2.00	2	4.00	1
2	4.00	2	4.00	2	4.00	2	4.00	2
1	5.00	2	4.00	4	2.00	1	5.00	2
2	4.00	2	4.00	3	3.00	2	4.00	2
3	3.00	6	#NULL!	4	2.00	2	4.00	4
4	2.00	4	2.00	3	3.00	2	4.00	3
1	5.00	1	5.00	1	5.00	1	5.00	3
4	2.00	2	4.00	2	4.00	2	4.00	5
4	2.00	4	2.00	3	3.00	3	3.00	2
4	2.00	3	3.00	2	4.00	2	4.00	4
4	2.00	2	4.00	2	4.00	2	4.00	4
3	3.00	3	3.00	2	4.00	2	4.00	4
2	4.00	2	4.00	2	4.00	3	3.00	2
2	4.00	3	3.00	2	4.00	2	4.00	4
4	2.00	4	2.00	4	2.00	2	4.00	5
2	4.00	2	4.00	2	4.00	2	4.00	3
4	2.00	2	4.00	4	2.00	2	4.00	1
4	2.00	4	2.00	2	4.00	1	5.00	2
3	3.00	3	3.00	3	3.00	3	3.00	3
3	3.00	5	1.00	5	1.00	2	4.00	2
4	2.00	3	3.00	2	4.00	2	4.00	4
4	2.00	4	2.00	4	2.00	2	4.00	5
5	1.00	5	1.00	5	1.00	1	5.00	5
4	2.00	5	1.00	2	4.00	1	5.00	4
5	1.00	5	1.00	2	4.00	1	5.00	4
3	3.00	2	4.00	2	4.00	2	4.00	3
2	4.00	4	2.00	2	4.00	2	4.00	3
4	2.00	5	1.00	2	4.00	1	5.00	3
4	2.00	4	2.00	2	4.00	2	4.00	5
2	4.00	3	3.00	2	4.00	1	5.00	2
4	2.00	4	2.00	3	3.00	3	3.00	2
5	1.00	3	3.00	3	3.00	2	4.00	5
4	2.00	5	1.00	2	4.00	1	5.00	3
1	5.00	2	4.00	1	5.00	1	5.00	1
4	2.00	1	5.00	1	5.00	2	4.00	4
1	5.00	3	3.00	1	5.00	1	5.00	1
2	4.00	3	3.00	1	5.00	1	5.00	4
3	3.00	3	3.00	2	4.00	2	4.00	2
3	3.00	3	3.00	2	4.00	1	5.00	3
4	2.00	5	1.00	3	3.00	1	5.00	5
5	1.00	4	2.00	3	3.00	1	5.00	1
4	2.00	5	1.00	3	3.00	2	4.00	4
4	2.00	5	1.00	3	3.00	3	3.00	4
4	2.00	2	4.00	2	4.00	2	4.00	5

5	1.00	5	1.00	5	1.00	3	3.00	5
2	4.00	2	4.00	2	4.00	2	4.00	2
3	3.00	3	3.00	2	4.00	2	4.00	3
3	3.00	4	2.00	3	3.00	2	4.00	5
2	4.00	2	4.00	3	3.00	2	4.00	2
2	4.00	2	4.00	2	4.00	2	4.00	3
3	3.00	2	4.00	2	4.00	2	4.00	4
3	3.00	4	2.00	2	4.00	2	4.00	5
2	4.00	1	5.00	1	5.00	1	5.00	1
1	5.00	1	5.00	2	4.00	1	5.00	1
4	2.00	3	3.00	2	4.00	2	4.00	2
2	4.00	5	1.00	6	#NULL!	6	#NULL!	5
1	5.00	3	3.00	1	5.00	1	5.00	3
2	4.00	3	3.00	2	4.00	2	4.00	5
3	3.00	3	3.00	6	#NULL!	6	#NULL!	2
2	4.00	2	4.00	3	3.00	2	4.00	2
3	3.00	3	3.00	3	3.00	3	3.00	3
3	3.00	3	3.00	2	4.00	3	3.00	2
2	4.00	2	4.00	3	3.00	2	4.00	2
3	3.00	3	3.00	3	3.00	3	3.00	3
2	4.00	3	3.00	3	3.00	2	4.00	2
2	4.00	3	3.00	3	3.00	3	3.00	3
3	3.00	3	3.00	4	2.00	3	3.00	3
2	4.00	2	4.00	2	4.00	2	4.00	2
5	1.00	4	2.00	6	#NULL!	3	3.00	5
3	3.00	4	2.00	2	4.00	2	4.00	3
4	2.00	2	4.00	3	3.00	2	4.00	4
4	2.00	2	4.00	2	4.00	2	4.00	4
2	4.00	5	1.00	2	4.00	2	4.00	4
3	3.00	3	3.00	3	3.00	2	4.00	4
2	4.00	2	4.00	2	4.00	2	4.00	5
4	2.00	1	5.00	2	4.00	1	5.00	4
2	4.00	2	4.00	2	4.00	2	4.00	2
4	2.00	2	4.00	4	2.00	1	5.00	4
2	4.00	2	4.00	2	4.00	2	4.00	3
2	4.00	2	4.00	2	4.00	2	4.00	3
5	1.00	2	4.00	6	#NULL!	1	5.00	4
3	3.00	3	3.00	3	3.00	3	3.00	3
3	3.00	3	3.00	3	3.00	3	3.00	3
4	2.00	5	1.00	4	2.00	2	4.00	5
4	2.00	2	4.00	4	2.00	2	4.00	4
3	3.00	2	4.00	2	4.00	2	4.00	2
2	4.00	2	4.00	2	4.00	2	4.00	4
3	3.00	2	4.00	2	4.00	2	4.00	2
2	4.00	2	4.00	2	4.00	2	4.00	2
4	2.00	6	#NULL!	6	#NULL!	6	#NULL!	5
1	5.00	3	3.00	2	4.00	2	4.00	2
3	3.00	2	4.00	4	2.00	3	3.00	5
4	2.00	1	5.00	3	3.00	3	3.00	4
5	1.00	3	3.00	3	3.00	3	3.00	3

4	2.00	2	4.00	3	3.00	3	3.00	3
5	1.00	2	4.00	4	2.00	3	3.00	4
4	2.00	5	1.00	3	3.00	2	4.00	2
4	2.00	4	2.00	2	4.00	2	4.00	4
1	5.00	2	4.00	2	4.00	2	4.00	5
4	2.00	2	4.00	2	4.00	2	4.00	2
2	4.00	1	5.00	3	3.00	2	4.00	4
3	3.00	3	3.00	3	3.00	3	3.00	2
5	1.00	3	3.00	3	3.00	2	4.00	5
4	2.00	1	5.00	2	4.00	1	5.00	3
2	4.00	2	4.00	2	4.00	2	4.00	2
2	4.00	1	5.00	3	3.00	1	5.00	2
4	2.00	2	4.00	3	3.00	3	3.00	2
4	2.00	2	4.00	3	3.00	2	4.00	2
3	3.00	3	3.00	2	4.00	2	4.00	4
2	4.00	2	4.00	4	2.00	2	4.00	3
1	5.00	1	5.00	1	5.00	1	5.00	1
3	3.00	2	4.00	2	4.00	2	4.00	3
2	4.00	4	2.00	4	2.00	2	4.00	4
4	2.00	3	3.00	2	4.00	2	4.00	2
5	1.00	3	3.00	2	4.00	2	4.00	2
3	3.00	2	4.00	2	4.00	2	4.00	3
2	4.00	3	3.00	2	4.00	3	3.00	2
3	3.00	2	4.00	2	4.00	2	4.00	3
1	5.00	1	5.00	3	3.00	2	4.00	1
2	4.00	2	4.00	2	4.00	2	4.00	1
2	4.00	3	3.00	2	4.00	3	3.00	3
3	3.00	3	3.00	3	3.00	3	3.00	3
5	1.00	2	4.00	2	4.00	2	4.00	5
3	3.00	2	4.00	2	4.00	2	4.00	2
2	4.00	2	4.00	2	4.00	2	4.00	2
5	1.00	3	3.00	6	#NULL!	2	4.00	5
4	2.00	2	4.00	1	5.00	1	5.00	4
3	3.00	6	#NULL!	6	#NULL!	6	#NULL!	3
4	2.00	1	5.00	4	2.00	1	5.00	5
4	2.00	2	4.00	4	2.00	4	2.00	4
5	1.00	3	3.00	6	#NULL!	3	3.00	4
1	5.00	2	4.00	2	4.00	2	4.00	2
2	4.00	2	4.00	2	4.00	2	4.00	2
4	2.00	4	2.00	3	3.00	5	1.00	4
4	2.00	6	#NULL!	6	#NULL!	6	#NULL!	5
5	1.00	5	1.00	4	2.00	4	2.00	4
2	4.00	2	4.00	2	4.00	2	4.00	2
4	2.00	2	4.00	2	4.00	2	4.00	5

Social_com	Recommer	Recommer	Employme	actual_sat	Gender	Age	Age_Recoc	age_grp_d
5.00	2	4.00	2	4.00	1	1	1	1.00
3.00	2	4.00	2	4.00	2	1	1	1.00
1.00	5	1.00	5	1.00	1	1	1	1.00
5.00	1	5.00	1	5.00	1	1	1	1.00
2.00	3	3.00	2	4.00	1	1	1	1.00
5.00	2	4.00	2	4.00	2	1	1	1.00
4.00	2	4.00	2	4.00	1	1	1	1.00
4.00	3	3.00	2	4.00	2	1	1	1.00
4.00	2	4.00	2	4.00	2	1	1	1.00
2.00	2	4.00	2	4.00	2	1	1	1.00
3.00	2	4.00	2	4.00	2	1	1	1.00
3.00	1	5.00	2	4.00	1	1	1	1.00
1.00	3	3.00	1	5.00	1	1	1	1.00
4.00	3	3.00	3	3.00	2	1	1	1.00
2.00	2	4.00	2	4.00	1	1	1	1.00
2.00	2	4.00	2	4.00	1	1	1	1.00
2.00	2	4.00	2	4.00	1	1	1	1.00
4.00	2	4.00	2	4.00	1	1	1	1.00
2.00	3	3.00	3	3.00	1	2	2	0.00
1.00	4	2.00	5	1.00	1	2	2	0.00
3.00	3	3.00	2	4.00	1	2	2	0.00
5.00	2	4.00	2	4.00	2	2	2	0.00
4.00	2	4.00	1	5.00	2	2	2	0.00
3.00	3	3.00	3	3.00	1	2	2	0.00
4.00	2	4.00	2	4.00	1	2	2	0.00
2.00	3	3.00	3	3.00	1	2	2	0.00
1.00	4	2.00	3	3.00	1	2	2	0.00
1.00	5	1.00	5	1.00	2	2	2	0.00
2.00	4	2.00	2	4.00	1	2	2	0.00
2.00	5	1.00	4	2.00	2	2	2	0.00
3.00	2	4.00	2	4.00	2	2	2	0.00
3.00	3	3.00	2	4.00	1	2	2	0.00
3.00	5	1.00	4	2.00	1	2	2	0.00
1.00	3	3.00	4	2.00	1	2	2	0.00
4.00	1	5.00	1	5.00	1	2	2	0.00
4.00	5	1.00	5	1.00	1	2	2	0.00
1.00	3	3.00	3	3.00	1	2	2	0.00
3.00	4	2.00	3	3.00	1	2	2	0.00
5.00	2	4.00	2	4.00	2	2	2	0.00
2.00	2	4.00	1	5.00	2	2	2	0.00
5.00	1	5.00	1	5.00	1	2	2	0.00
2.00	2	4.00	2	4.00	2	2	2	0.00
4.00	2	4.00	2	4.00	1	2	2	0.00
3.00	2	4.00	2	4.00	1	2	2	0.00
1.00	3	3.00	3	3.00	2	2	2	0.00
5.00	5	1.00	4	2.00	1	2	2	0.00
2.00	4	2.00	3	3.00	1	2	2	0.00
2.00	3	3.00	2	4.00	1	2	2	0.00
1.00	5	1.00	4	2.00	1	3	3	0.00

1.00	5	1.00	5	1.00	1	3	3	0.00
4.00	1	5.00	1	5.00	2	3	3	0.00
3.00	3	3.00	3	3.00	2	3	3	0.00
1.00	3	3.00	3	3.00	1	3	3	0.00
4.00	2	4.00	2	4.00	1	3	3	0.00
3.00	3	3.00	2	4.00	2	3	3	0.00
2.00	3	3.00	3	3.00	1	3	3	0.00
1.00	3	3.00	3	3.00	1	3	3	0.00
5.00	1	5.00	1	5.00	2	4	3	0.00
5.00	2	4.00	1	5.00	1	4	3	0.00
4.00	2	4.00	2	4.00	1	4	3	0.00
1.00	3	3.00	5	1.00	1	1	1	1.00
3.00	1	5.00	3	3.00	1	1	1	1.00
1.00	3	3.00	3	3.00	1	1	1	1.00
4.00	2	4.00	1	5.00	1	1	1	1.00
4.00	2	4.00	4	2.00	2	1	1	1.00
3.00	3	3.00	3	3.00	2	1	1	1.00
4.00	2	4.00	3	3.00	2	1	1	1.00
4.00	2	4.00	2	4.00	1	2	2	0.00
3.00	3	3.00	2	4.00	1	2	2	0.00
4.00	2	4.00	2	4.00	1	2	2	0.00
3.00	3	3.00	3	3.00	1	2	2	0.00
3.00	3	3.00	3	3.00	1	2	2	0.00
4.00	2	4.00	2	4.00	1	2	2	0.00
1.00	4	2.00	3	3.00	1	2	2	0.00
3.00	3	3.00	1	5.00	1	2	2	0.00
2.00	2	4.00	2	4.00	1	2	2	0.00
2.00	4	2.00	2	4.00	2	2	2	0.00
2.00	2	4.00	2	4.00	1	2	2	0.00
2.00	2	4.00	1	5.00	1	2	2	0.00
1.00	4	2.00	4	2.00	1	2	2	0.00
2.00	1	5.00	1	5.00	1	2	2	0.00
4.00	1	5.00	1	5.00	1	2	2	0.00
2.00	4	2.00	4	2.00	1	2	2	0.00
3.00	2	4.00	2	4.00	1	2	2	0.00
3.00	2	4.00	1	5.00	1	2	2	0.00
2.00	3	3.00	2	4.00	1	2	2	0.00
3.00	3	3.00	3	3.00	1	2	2	0.00
3.00	3	3.00	3	3.00	1	2	2	0.00
1.00	2	4.00	2	4.00	1	2	2	0.00
2.00	2	4.00	1	5.00	1	2	2	0.00
4.00	2	4.00	2	4.00	1	2	2	0.00
2.00	3	3.00	2	4.00	1	2	2	0.00
4.00	1	5.00	3	3.00	1	2	2	0.00
4.00	2	4.00	2	4.00	1	2	2	0.00
1.00	5	1.00	5	1.00	1	2	2	0.00
4.00	2	4.00	2	4.00	1	2	2	0.00
1.00	3	3.00	3	3.00	1	2	2	0.00
2.00	3	3.00	3	3.00	1	2	2	0.00
3.00	5	1.00	4	2.00	1	2	2	0.00

3.00	2	4.00	2	4.00	1	2	2	0.00
2.00	3	3.00	2	4.00	1	2	2	0.00
4.00	4	2.00	5	1.00	1	2	2	0.00
2.00	2	4.00	2	4.00	1	2	2	0.00
1.00	2	4.00	2	4.00	1	3	3	0.00
4.00	2	4.00	2	4.00	1	3	3	0.00
2.00	2	4.00	2	4.00	1	3	3	0.00
4.00	2	4.00	3	3.00	1	3	3	0.00
1.00	3	3.00	3	3.00	1	3	3	0.00
3.00	3	3.00	2	4.00	1	3	3	0.00
4.00	2	4.00	2	4.00	1	3	3	0.00
4.00	2	4.00	2	4.00	2	3	3	0.00
4.00	2	4.00	1	5.00	1	3	3	0.00
4.00	2	4.00	1	5.00	1	3	3	0.00
2.00	2	4.00	2	4.00	1	3	3	0.00
3.00	2	4.00	2	4.00	1	4	3	0.00
5.00	1	5.00	1	5.00	1	4	3	0.00
3.00	3	3.00	2	4.00	1	4	3	0.00
2.00	4	2.00	4	2.00	1	1	1	1.00
4.00	3	3.00	2	4.00	1	1	1	1.00
4.00	3	3.00	3	3.00	1	3	3	0.00
3.00	3	3.00	3	3.00	1	2	2	0.00
4.00	3	3.00	3	3.00	1	3	3	0.00
3.00	3	3.00	2	4.00	1	1	1	1.00
5.00	1	5.00	2	4.00	2	2	2	0.00
5.00	2	4.00	2	4.00	2	2	2	0.00
3.00	1	5.00	3	3.00	1	3	3	0.00
3.00	2	4.00	3	3.00	1	3	3	0.00
1.00	5	1.00	4	2.00	1	3	3	0.00
4.00	3	3.00	3	3.00	1	1	1	1.00
4.00	1	5.00	1	5.00	2	2	2	0.00
1.00	5	1.00	5	1.00	2	2	2	0.00
2.00	3	3.00	2	4.00	2	3	3	0.00
3.00	2	4.00	2	4.00	1	3	3	0.00
1.00	2	4.00	1	5.00	2	3	3	0.00
2.00	3	3.00	2	4.00	2	3	3	0.00
2.00	4	2.00	5	1.00	1	1	1	1.00
4.00	2	4.00	2	4.00	1	1	1	1.00
4.00	3	3.00	2	4.00	1	1	1	1.00
2.00	5	1.00	5	1.00	1	1	1	1.00
1.00	5	1.00	5	1.00	1	2	2	0.00
2.00	5	1.00	5	1.00	2	2	2	0.00
4.00	2	4.00	2	4.00	1	2	2	0.00
1.00	5	1.00	4	2.00	1	3	3	0.00

age_grp_d	age_grp_d	Marital_sta	Marital_sta	MS_grp_d	MS_grp_d	MS_grp_d	Educationa	Educationa
0.00	0.00	2	2	0.00	1.00	0.00	2	2
0.00	0.00	2	2	0.00	1.00	0.00	2	2
0.00	0.00	1	1	1.00	0.00	0.00	2	2
0.00	0.00	1	1	1.00	0.00	0.00	2	2
0.00	0.00	1	1	1.00	0.00	0.00	2	2
0.00	0.00	2	2	0.00	1.00	0.00	2	2
0.00	0.00	2	2	0.00	1.00	0.00	4	3
0.00	0.00	1	1	1.00	0.00	0.00	2	2
0.00	0.00	1	1	1.00	0.00	0.00	2	2
0.00	0.00	1	1	1.00	0.00	0.00	2	2
0.00	0.00	1	1	1.00	0.00	0.00	2	2
0.00	0.00	1	1	1.00	0.00	0.00	2	2
0.00	0.00	2	2	0.00	1.00	0.00	2	2
0.00	0.00	1	1	1.00	0.00	0.00	2	2
0.00	0.00	2	2	0.00	1.00	0.00	2	2
0.00	0.00	2	2	0.00	1.00	0.00	2	2
0.00	0.00	2	2	0.00	1.00	0.00	2	2
0.00	0.00	3	3	0.00	0.00	1.00	2	2
1.00	0.00	2	2	0.00	1.00	0.00	2	2
1.00	0.00	2	2	0.00	1.00	0.00	1	1
1.00	0.00	2	2	0.00	1.00	0.00	4	3
1.00	0.00	2	2	0.00	1.00	0.00	4	3
1.00	0.00	2	2	0.00	1.00	0.00	2	2
1.00	0.00	4	3	0.00	0.00	1.00	2	2
1.00	0.00	1	1	1.00	0.00	0.00	2	2
1.00	0.00	2	2	0.00	1.00	0.00	2	2
1.00	0.00	2	2	0.00	1.00	0.00	4	3
1.00	0.00	2	2	0.00	1.00	0.00	2	2
1.00	0.00	2	2	0.00	1.00	0.00	3	3
1.00	0.00	1	1	1.00	0.00	0.00	4	3
1.00	0.00	2	2	0.00	1.00	0.00	4	3
1.00	0.00	2	2	0.00	1.00	0.00	2	2
1.00	0.00	1	1	1.00	0.00	0.00	2	2
1.00	0.00	2	2	0.00	1.00	0.00	4	3
1.00	0.00	1	1	1.00	0.00	0.00	4	3
1.00	0.00	2	2	0.00	1.00	0.00	2	2
1.00	0.00	2	2	0.00	1.00	0.00	2	2
1.00	0.00	2	2	0.00	1.00	0.00	2	2
1.00	0.00	2	2	0.00	1.00	0.00	2	2
1.00	0.00	2	2	0.00	1.00	0.00	2	2
1.00	0.00	2	2	0.00	1.00	0.00	4	3
1.00	0.00	2	2	0.00	1.00	0.00	2	2
1.00	0.00	3	3	0.00	0.00	1.00	2	2
1.00	0.00	2	2	0.00	1.00	0.00	2	2
1.00	0.00	2	2	0.00	1.00	0.00	2	2
1.00	0.00	2	2	0.00	1.00	0.00	3	3
1.00	0.00	2	2	0.00	1.00	0.00	4	3
1.00	0.00	2	2	0.00	1.00	0.00	2	2
0.00	1.00	2	2	0.00	1.00	0.00	2	2

0.00	1.00	2	2	0.00	1.00	0.00	2	2
0.00	1.00	2	2	0.00	1.00	0.00	2	2
0.00	1.00	2	2	0.00	1.00	0.00	4	3
0.00	1.00	2	2	0.00	1.00	0.00	3	3
0.00	1.00	2	2	0.00	1.00	0.00	2	2
0.00	1.00	2	2	0.00	1.00	0.00	1	1
0.00	1.00	3	3	0.00	0.00	1.00	2	2
0.00	1.00	3	3	0.00	0.00	1.00	2	2
0.00	1.00	2	2	0.00	1.00	0.00	2	2
0.00	1.00	2	2	0.00	1.00	0.00	2	2
0.00	1.00	2	2	0.00	1.00	0.00	2	2
0.00	0.00	1	1	1.00	0.00	0.00	2	2
0.00	0.00	1	1	1.00	0.00	0.00	2	2
0.00	0.00	2	2	0.00	1.00	0.00	1	1
0.00	0.00	1	1	1.00	0.00	0.00	1	1
0.00	0.00	1	1	1.00	0.00	0.00	2	2
0.00	0.00	1	1	1.00	0.00	0.00	2	2
0.00	0.00	1	1	1.00	0.00	0.00	2	2
1.00	0.00	2	2	0.00	1.00	0.00	1	1
1.00	0.00	2	2	0.00	1.00	0.00	1	1
1.00	0.00	2	2	0.00	1.00	0.00	1	1
1.00	0.00	2	2	0.00	1.00	0.00	1	1
1.00	0.00	2	2	0.00	1.00	0.00	1	1
1.00	0.00	1	1	1.00	0.00	0.00	1	1
1.00	0.00	2	2	0.00	1.00	0.00	1	1
1.00	0.00	2	2	0.00	1.00	0.00	1	1
1.00	0.00	1	1	1.00	0.00	0.00	1	1
1.00	0.00	2	2	0.00	1.00	0.00	1	1
1.00	0.00	2	2	0.00	1.00	0.00	2	2
1.00	0.00	2	2	0.00	1.00	0.00	1	1
1.00	0.00	2	2	0.00	1.00	0.00	1	1
1.00	0.00	2	2	0.00	1.00	0.00	1	1
1.00	0.00	2	2	0.00	1.00	0.00	1	1
1.00	0.00	1	1	1.00	0.00	0.00	1	1
1.00	0.00	3	3	0.00	0.00	1.00	1	1
1.00	0.00	2	2	0.00	1.00	0.00	2	2
1.00	0.00	2	2	0.00	1.00	0.00	1	1
1.00	0.00	2	2	0.00	1.00	0.00	2	2
1.00	0.00	2	2	0.00	1.00	0.00	2	2
1.00	0.00	2	2	0.00	1.00	0.00	1	1
1.00	0.00	2	2	0.00	1.00	0.00	2	2
1.00	0.00	2	2	0.00	1.00	0.00	1	1
1.00	0.00	2	2	0.00	1.00	0.00	1	1
1.00	0.00	2	2	0.00	1.00	0.00	1	1
1.00	0.00	2	2	0.00	1.00	0.00	1	1
1.00	0.00	2	2	0.00	1.00	0.00	2	2
1.00	0.00	2	2	0.00	1.00	0.00	1	1
1.00	0.00	2	2	0.00	1.00	0.00	2	2
1.00	0.00	3	3	0.00	0.00	1.00	2	2
1.00	0.00	2	2	0.00	1.00	0.00	2	2

1.00	0.00	2	2	0.00	1.00	0.00	1	1
1.00	0.00	2	2	0.00	1.00	0.00	1	1
1.00	0.00	1	1	1.00	0.00	0.00	2	2
1.00	0.00	2	2	0.00	1.00	0.00	3	3
0.00	1.00	3	3	0.00	0.00	1.00	1	1
0.00	1.00	1	1	1.00	0.00	0.00	1	1
0.00	1.00	2	2	0.00	1.00	0.00	1	1
0.00	1.00	2	2	0.00	1.00	0.00	1	1
0.00	1.00	2	2	0.00	1.00	0.00	2	2
0.00	1.00	3	3	0.00	0.00	1.00	1	1
0.00	1.00	2	2	0.00	1.00	0.00	1	1
0.00	1.00	2	2	0.00	1.00	0.00	1	1
0.00	1.00	1	1	1.00	0.00	0.00	1	1
0.00	1.00	2	2	0.00	1.00	0.00	1	1
0.00	1.00	2	2	0.00	1.00	0.00	1	1
0.00	1.00	2	2	0.00	1.00	0.00	2	2
0.00	1.00	2	2	0.00	1.00	0.00	1	1
0.00	1.00	2	2	0.00	1.00	0.00	1	1
0.00	0.00	2	2	0.00	1.00	0.00	2	2
0.00	0.00	1	1	1.00	0.00	0.00	2	2
0.00	1.00	2	2	0.00	1.00	0.00	1	1
1.00	0.00	2	2	0.00	1.00	0.00	2	2
0.00	1.00	2	2	0.00	1.00	0.00	2	2
0.00	0.00	2	2	0.00	1.00	0.00	2	2
1.00	0.00	2	2	0.00	1.00	0.00	2	2
1.00	0.00	2	2	0.00	1.00	0.00	3	3
0.00	1.00	2	2	0.00	1.00	0.00	1	1
0.00	1.00	2	2	0.00	1.00	0.00	2	2
0.00	1.00	3	3	0.00	0.00	1.00	3	3
0.00	0.00	1	1	1.00	0.00	0.00	2	2
1.00	0.00	2	2	0.00	1.00	0.00	1	1
1.00	0.00	2	2	0.00	1.00	0.00	2	2
0.00	1.00	2	2	0.00	1.00	0.00	1	1
0.00	1.00	2	2	0.00	1.00	0.00	4	3
0.00	1.00	2	2	0.00	1.00	0.00	1	1
0.00	1.00	2	2	0.00	1.00	0.00	1	1
0.00	0.00	3	3	0.00	0.00	1.00	3	3
0.00	0.00	2	2	0.00	1.00	0.00	3	3
0.00	0.00	2	2	0.00	1.00	0.00	2	2
0.00	0.00	2	2	0.00	1.00	0.00	3	3
1.00	0.00	2	2	0.00	1.00	0.00	2	2
1.00	0.00	2	2	0.00	1.00	0.00	2	2
1.00	0.00	2	2	0.00	1.00	0.00	2	2
0.00	1.00	2	2	0.00	1.00	0.00	2	2

Edu_grp_d	Edu_grp_d	Edu_grp_d	Specialty	Specialty_c	Speciality_	sp_grp_d_	sp_grp_d_	sp_grp_d_
0.00	1.00	0.00	1	emergency	1	1.00	0.00	0.00
0.00	1.00	0.00	1		1	1.00	0.00	0.00
0.00	1.00	0.00	1		1	1.00	0.00	0.00
0.00	1.00	0.00	1		1	1.00	0.00	0.00
0.00	1.00	0.00	1		1	1.00	0.00	0.00
0.00	1.00	0.00	1		1	1.00	0.00	0.00
0.00	0.00	1.00	1		1	1.00	0.00	0.00
0.00	1.00	0.00	1		1	1.00	0.00	0.00
0.00	1.00	0.00	1		1	1.00	0.00	0.00
0.00	1.00	0.00	1		1	1.00	0.00	0.00
0.00	1.00	0.00	1		1	1.00	0.00	0.00
0.00	1.00	0.00	1		1	1.00	0.00	0.00
0.00	1.00	0.00	1		1	1.00	0.00	0.00
0.00	1.00	0.00	1		1	1.00	0.00	0.00
0.00	1.00	0.00	1		1	1.00	0.00	0.00
0.00	1.00	0.00	1		1	1.00	0.00	0.00
0.00	1.00	0.00	1		1	1.00	0.00	0.00
0.00	1.00	0.00	1		1	1.00	0.00	0.00
0.00	1.00	0.00	1	Dentist	1	1.00	0.00	0.00
1.00	0.00	0.00	1	Dentist	1	1.00	0.00	0.00
0.00	0.00	1.00	1		1	1.00	0.00	0.00
0.00	0.00	1.00	1		1	1.00	0.00	0.00
0.00	1.00	0.00	1		1	1.00	0.00	0.00
0.00	1.00	0.00	1		1	1.00	0.00	0.00
0.00	1.00	0.00	1		1	1.00	0.00	0.00
0.00	1.00	0.00	1		1	1.00	0.00	0.00
0.00	0.00	1.00	1		1	1.00	0.00	0.00
0.00	1.00	0.00	1		1	1.00	0.00	0.00
0.00	0.00	1.00	1		1	1.00	0.00	0.00
0.00	0.00	1.00	1		1	1.00	0.00	0.00
0.00	0.00	1.00	1		1	1.00	0.00	0.00
0.00	1.00	0.00	1		1	1.00	0.00	0.00
0.00	1.00	0.00	1		1	1.00	0.00	0.00
0.00	0.00	1.00	1		1	1.00	0.00	0.00
0.00	0.00	1.00	1		1	1.00	0.00	0.00
0.00	1.00	0.00	1		1	1.00	0.00	0.00
0.00	1.00	0.00	1		1	1.00	0.00	0.00
0.00	1.00	0.00	1		1	1.00	0.00	0.00
0.00	1.00	0.00	1		1	1.00	0.00	0.00
0.00	1.00	0.00	1		1	1.00	0.00	0.00
0.00	0.00	1.00	1		1	1.00	0.00	0.00
0.00	1.00	0.00	1		1	1.00	0.00	0.00
0.00	1.00	0.00	1		1	1.00	0.00	0.00
0.00	1.00	0.00	1		1	1.00	0.00	0.00
0.00	1.00	0.00	1		1	1.00	0.00	0.00
0.00	0.00	1.00	1		1	1.00	0.00	0.00
0.00	0.00	1.00	1		1	1.00	0.00	0.00
0.00	1.00	0.00	1		1	1.00	0.00	0.00
0.00	1.00	0.00	1		1	1.00	0.00	0.00

0.00	1.00	0.00	1		1	1.00	0.00	0.00
0.00	1.00	0.00	1		1	1.00	0.00	0.00
0.00	0.00	1.00	1		1	1.00	0.00	0.00
0.00	0.00	1.00	1		1	1.00	0.00	0.00
0.00	1.00	0.00	1		1	1.00	0.00	0.00
1.00	0.00	0.00	1		1	1.00	0.00	0.00
0.00	1.00	0.00	1		1	1.00	0.00	0.00
0.00	1.00	0.00	1		1	1.00	0.00	0.00
0.00	1.00	0.00	1	GP	1	1.00	0.00	0.00
0.00	1.00	0.00	1		1	1.00	0.00	0.00
0.00	1.00	0.00	1		1	1.00	0.00	0.00
0.00	1.00	0.00	2		2	0.00	1.00	0.00
0.00	1.00	0.00	2		2	0.00	1.00	0.00
1.00	0.00	0.00	2		2	0.00	1.00	0.00
1.00	0.00	0.00	2		2	0.00	1.00	0.00
0.00	1.00	0.00	2		2	0.00	1.00	0.00
0.00	1.00	0.00	2		2	0.00	1.00	0.00
0.00	1.00	0.00	2		2	0.00	1.00	0.00
1.00	0.00	0.00	2	nurse	2	0.00	1.00	0.00
1.00	0.00	0.00	2	assisstante	2	0.00	1.00	0.00
1.00	0.00	0.00	2		2	0.00	1.00	0.00
1.00	0.00	0.00	2		2	0.00	1.00	0.00
1.00	0.00	0.00	2		2	0.00	1.00	0.00
1.00	0.00	0.00	2		2	0.00	1.00	0.00
1.00	0.00	0.00	2		2	0.00	1.00	0.00
1.00	0.00	0.00	2		2	0.00	1.00	0.00
1.00	0.00	0.00	2		2	0.00	1.00	0.00
1.00	0.00	0.00	2		2	0.00	1.00	0.00
0.00	1.00	0.00	2		2	0.00	1.00	0.00
1.00	0.00	0.00	2		2	0.00	1.00	0.00
1.00	0.00	0.00	2		2	0.00	1.00	0.00
1.00	0.00	0.00	2		2	0.00	1.00	0.00
1.00	0.00	0.00	2		2	0.00	1.00	0.00
1.00	0.00	0.00	2		2	0.00	1.00	0.00
1.00	0.00	0.00	2		2	0.00	1.00	0.00
0.00	1.00	0.00	2		2	0.00	1.00	0.00
1.00	0.00	0.00	2		2	0.00	1.00	0.00
0.00	1.00	0.00	2		2	0.00	1.00	0.00
0.00	1.00	0.00	2		2	0.00	1.00	0.00
1.00	0.00	0.00	2		2	0.00	1.00	0.00
0.00	1.00	0.00	2		2	0.00	1.00	0.00
1.00	0.00	0.00	2		2	0.00	1.00	0.00
1.00	0.00	0.00	2		2	0.00	1.00	0.00
1.00	0.00	0.00	2		2	0.00	1.00	0.00
1.00	0.00	0.00	2		2	0.00	1.00	0.00
0.00	1.00	0.00	2		2	0.00	1.00	0.00
1.00	0.00	0.00	2		2	0.00	1.00	0.00
0.00	1.00	0.00	2		2	0.00	1.00	0.00
0.00	1.00	0.00	2		2	0.00	1.00	0.00
0.00	1.00	0.00	2		2	0.00	1.00	0.00

1.00	0.00	0.00	2		2	0.00	1.00	0.00
1.00	0.00	0.00	2		2	0.00	1.00	0.00
0.00	1.00	0.00	2		2	0.00	1.00	0.00
0.00	0.00	1.00	2		2	0.00	1.00	0.00
1.00	0.00	0.00	2	assisstante	2	0.00	1.00	0.00
1.00	0.00	0.00	2	Nurse	2	0.00	1.00	0.00
1.00	0.00	0.00	2		2	0.00	1.00	0.00
1.00	0.00	0.00	2		2	0.00	1.00	0.00
0.00	1.00	0.00	2		2	0.00	1.00	0.00
1.00	0.00	0.00	2		2	0.00	1.00	0.00
1.00	0.00	0.00	2		2	0.00	1.00	0.00
1.00	0.00	0.00	2		2	0.00	1.00	0.00
1.00	0.00	0.00	2		2	0.00	1.00	0.00
1.00	0.00	0.00	2		2	0.00	1.00	0.00
1.00	0.00	0.00	2		2	0.00	1.00	0.00
0.00	1.00	0.00	2	midwife	2	0.00	1.00	0.00
1.00	0.00	0.00	2		2	0.00	1.00	0.00
1.00	0.00	0.00	2		2	0.00	1.00	0.00
0.00	1.00	0.00	3		3	0.00	0.00	1.00
0.00	1.00	0.00	3		3	0.00	0.00	1.00
1.00	0.00	0.00	3		3	0.00	0.00	1.00
0.00	1.00	0.00	5	Allied heal	3	0.00	0.00	1.00
0.00	1.00	0.00	4	Tech	3	0.00	0.00	1.00
0.00	1.00	0.00	4		3	0.00	0.00	1.00
0.00	1.00	0.00	4		3	0.00	0.00	1.00
0.00	0.00	1.00	4		3	0.00	0.00	1.00
1.00	0.00	0.00	4		3	0.00	0.00	1.00
0.00	1.00	0.00	4		3	0.00	0.00	1.00
0.00	0.00	1.00	4		3	0.00	0.00	1.00
0.00	1.00	0.00	5		3	0.00	0.00	1.00
1.00	0.00	0.00	5	radiologist	3	0.00	0.00	1.00
0.00	1.00	0.00	5		3	0.00	0.00	1.00
1.00	0.00	0.00	5		3	0.00	0.00	1.00
0.00	0.00	1.00	5		3	0.00	0.00	1.00
1.00	0.00	0.00	5		3	0.00	0.00	1.00
1.00	0.00	0.00	5		3	0.00	0.00	1.00
0.00	0.00	1.00	6	Public Hea	4	0.00	0.00	0.00
0.00	0.00	1.00	6	Public Hea	4	0.00	0.00	0.00
0.00	1.00	0.00	6	Public Hea	4	0.00	0.00	0.00
0.00	0.00	1.00	6	Public Hea	4	0.00	0.00	0.00
0.00	1.00	0.00	6	Public Hea	4	0.00	0.00	0.00
0.00	1.00	0.00	6	Public Hea	4	0.00	0.00	0.00
0.00	1.00	0.00	6	Public Hea	4	0.00	0.00	0.00
0.00	1.00	0.00	6	Public Hea	4	0.00	0.00	0.00

sp_grp_d_	Years_exp	year_grp_	year_grp_	year_grp_	year_grp_	Salaryrang	sal_grp_d_	sal_grp_d_
0.00	2	0.00	1.00	0.00	0.00	2	0.00	1.00
0.00	2	0.00	1.00	0.00	0.00	2	0.00	1.00
0.00	1	1.00	0.00	0.00	0.00	2	0.00	1.00
0.00	3	0.00	0.00	1.00	0.00	2	0.00	1.00
0.00	1	1.00	0.00	0.00	0.00	2	0.00	1.00
0.00	2	0.00	1.00	0.00	0.00	1	1.00	0.00
0.00	2	0.00	1.00	0.00	0.00	2	0.00	1.00
0.00	2	0.00	1.00	0.00	0.00	2	0.00	1.00
0.00	1	1.00	0.00	0.00	0.00	3	0.00	0.00
0.00	1	1.00	0.00	0.00	0.00	3	0.00	0.00
0.00	1	1.00	0.00	0.00	0.00	3	0.00	0.00
0.00	1	1.00	0.00	0.00	0.00	2	0.00	1.00
0.00	2	0.00	1.00	0.00	0.00	2	0.00	1.00
0.00	1	1.00	0.00	0.00	0.00	2	0.00	1.00
0.00	2	0.00	1.00	0.00	0.00	2	0.00	1.00
0.00	2	0.00	1.00	0.00	0.00	2	0.00	1.00
0.00	2	0.00	1.00	0.00	0.00	2	0.00	1.00
0.00	1	1.00	0.00	0.00	0.00	2	0.00	1.00
0.00	4	0.00	0.00	0.00	1.00	3	0.00	0.00
0.00	2	0.00	1.00	0.00	0.00	1	1.00	0.00
0.00	4	0.00	0.00	0.00	1.00	3	0.00	0.00
0.00	3	0.00	0.00	1.00	0.00	2	0.00	1.00
0.00	3	0.00	0.00	1.00	0.00	3	0.00	0.00
0.00	4	0.00	0.00	0.00	1.00	3	0.00	0.00
0.00	4	0.00	0.00	0.00	1.00	3	0.00	0.00
0.00	4	0.00	0.00	0.00	1.00	3	0.00	0.00
0.00	2	0.00	1.00	0.00	0.00	3	0.00	0.00
0.00	3	0.00	0.00	1.00	0.00	3	0.00	0.00
0.00	4	0.00	0.00	0.00	1.00	3	0.00	0.00
0.00	2	0.00	1.00	0.00	0.00	2	0.00	1.00
0.00	3	0.00	0.00	1.00	0.00	3	0.00	0.00
0.00	3	0.00	0.00	1.00	0.00	3	0.00	0.00
0.00	3	0.00	0.00	1.00	0.00	3	0.00	0.00
0.00	3	0.00	0.00	1.00	0.00	3	0.00	0.00
0.00	3	0.00	0.00	1.00	0.00	3	0.00	0.00
0.00	3	0.00	0.00	1.00	0.00	3	0.00	0.00
0.00	4	0.00	0.00	0.00	1.00	3	0.00	0.00
0.00	4	0.00	0.00	0.00	1.00	3	0.00	0.00
0.00	2	0.00	1.00	0.00	0.00	3	0.00	0.00
0.00	4	0.00	0.00	0.00	1.00	3	0.00	0.00
0.00	4	0.00	0.00	0.00	1.00	3	0.00	0.00
0.00	4	0.00	0.00	0.00	1.00	3	0.00	0.00
0.00	3	0.00	0.00	1.00	0.00	3	0.00	0.00
0.00	4	0.00	0.00	0.00	1.00	2	0.00	1.00
0.00	4	0.00	0.00	0.00	1.00	2	0.00	1.00
0.00	2	0.00	1.00	0.00	0.00	3	0.00	0.00
0.00	3	0.00	0.00	1.00	0.00	3	0.00	0.00
0.00	4	0.00	0.00	0.00	1.00	2	0.00	1.00
0.00	4	0.00	0.00	0.00	1.00	3	0.00	0.00

0.00	4	0.00	0.00	0.00	1.00	3	0.00	0.00
0.00	4	0.00	0.00	0.00	1.00	1	1.00	0.00
0.00	4	0.00	0.00	0.00	1.00	3	0.00	0.00
0.00	3	0.00	0.00	1.00	0.00	3	0.00	0.00
0.00	3	0.00	0.00	1.00	0.00	1	1.00	0.00
0.00	4	0.00	0.00	0.00	1.00	2	0.00	1.00
0.00	4	0.00	0.00	0.00	1.00	1	1.00	0.00
0.00	4	0.00	0.00	0.00	1.00	2	0.00	1.00
0.00	4	0.00	0.00	0.00	1.00	1	1.00	0.00
0.00	4	0.00	0.00	0.00	1.00	2	0.00	1.00
0.00	4	0.00	0.00	0.00	1.00	2	0.00	1.00
0.00	1	1.00	0.00	0.00	0.00	1	1.00	0.00
0.00	2	0.00	1.00	0.00	0.00	1	1.00	0.00
0.00	2	0.00	1.00	0.00	0.00	1	1.00	0.00
0.00	4	0.00	0.00	0.00	1.00	2	0.00	1.00
0.00	3	0.00	0.00	1.00	0.00	2	0.00	1.00
0.00	1	1.00	0.00	0.00	0.00	1	1.00	0.00
0.00	1	1.00	0.00	0.00	0.00	1	1.00	0.00
0.00	4	0.00	0.00	0.00	1.00	2	0.00	1.00
0.00	2	0.00	1.00	0.00	0.00	2	0.00	1.00
0.00	4	0.00	0.00	0.00	1.00	2	0.00	1.00
0.00	2	0.00	1.00	0.00	0.00	2	0.00	1.00
0.00	4	0.00	0.00	0.00	1.00	2	0.00	1.00
0.00	4	0.00	0.00	0.00	1.00	2	0.00	1.00
0.00	4	0.00	0.00	0.00	1.00	2	0.00	1.00
0.00	2	0.00	1.00	0.00	0.00	2	0.00	1.00
0.00	4	0.00	0.00	0.00	1.00	2	0.00	1.00
0.00	4	0.00	0.00	0.00	1.00	1	1.00	0.00
0.00	4	0.00	0.00	0.00	1.00	2	0.00	1.00
0.00	4	0.00	0.00	0.00	1.00	2	0.00	1.00
0.00	4	0.00	0.00	0.00	1.00	2	0.00	1.00
0.00	4	0.00	0.00	0.00	1.00	2	0.00	1.00
0.00	4	0.00	0.00	0.00	1.00	2	0.00	1.00
0.00	4	0.00	0.00	0.00	1.00	1	1.00	0.00
0.00	4	0.00	0.00	0.00	1.00	2	0.00	1.00
0.00	4	0.00	0.00	0.00	1.00	2	0.00	1.00
0.00	4	0.00	0.00	0.00	1.00	2	0.00	1.00
0.00	4	0.00	0.00	0.00	1.00	2	0.00	1.00
0.00	4	0.00	0.00	0.00	1.00	1	1.00	0.00
0.00	4	0.00	0.00	0.00	1.00	1	1.00	0.00
0.00	4	0.00	0.00	0.00	1.00	2	0.00	1.00
0.00	4	0.00	0.00	0.00	1.00	1	1.00	0.00
0.00	2	0.00	1.00	0.00	0.00	2	0.00	1.00
0.00	4	0.00	0.00	0.00	1.00	2	0.00	1.00
0.00	4	0.00	0.00	0.00	1.00	1	1.00	0.00
0.00	4	0.00	0.00	0.00	1.00	2	0.00	1.00
0.00	4	0.00	0.00	0.00	1.00	2	0.00	1.00
0.00	3	0.00	0.00	1.00	0.00	2	0.00	1.00
0.00	4	0.00	0.00	0.00	1.00	2	0.00	1.00
0.00	4	0.00	0.00	0.00	1.00	2	0.00	1.00

0.00	4	0.00	0.00	0.00	1.00	2	0.00	1.00
0.00	4	0.00	0.00	0.00	1.00	2	0.00	1.00
0.00	3	0.00	0.00	1.00	0.00	2	0.00	1.00
0.00	4	0.00	0.00	0.00	1.00	2	0.00	1.00
0.00	4	0.00	0.00	0.00	1.00	2	0.00	1.00
0.00	4	0.00	0.00	0.00	1.00	1	1.00	0.00
0.00	4	0.00	0.00	0.00	1.00	2	0.00	1.00
0.00	4	0.00	0.00	0.00	1.00	2	0.00	1.00
0.00	4	0.00	0.00	0.00	1.00	2	0.00	1.00
0.00	4	0.00	0.00	0.00	1.00	2	0.00	1.00
0.00	4	0.00	0.00	0.00	1.00	2	0.00	1.00
0.00	4	0.00	0.00	0.00	1.00	2	0.00	1.00
0.00	4	0.00	0.00	0.00	1.00	2	0.00	1.00
0.00	4	0.00	0.00	0.00	1.00	2	0.00	1.00
0.00	4	0.00	0.00	0.00	1.00	2	0.00	1.00
0.00	4	0.00	0.00	0.00	1.00	2	0.00	1.00
0.00	4	0.00	0.00	0.00	1.00	3	0.00	0.00
0.00	4	0.00	0.00	0.00	1.00	3	0.00	0.00
0.00	2	0.00	1.00	0.00	0.00	2	0.00	1.00
0.00	2	0.00	1.00	0.00	0.00	2	0.00	1.00
0.00	4	0.00	0.00	0.00	1.00	2	0.00	1.00
0.00	4	0.00	0.00	0.00	1.00	2	0.00	1.00
0.00	4	0.00	0.00	0.00	1.00	1	1.00	0.00
0.00	3	0.00	0.00	1.00	0.00	2	0.00	1.00
0.00	3	0.00	0.00	1.00	0.00	1	1.00	0.00
0.00	4	0.00	0.00	0.00	1.00	2	0.00	1.00
0.00	4	0.00	0.00	0.00	1.00	3	0.00	0.00
0.00	3	0.00	0.00	1.00	0.00	1	1.00	0.00
0.00	4	0.00	0.00	0.00	1.00	3	0.00	0.00
0.00	3	0.00	0.00	1.00	0.00	1	1.00	0.00
0.00	3	0.00	0.00	1.00	0.00	2	0.00	1.00
0.00	4	0.00	0.00	0.00	1.00	2	0.00	1.00
0.00	4	0.00	0.00	0.00	1.00	2	0.00	1.00
0.00	3	0.00	0.00	1.00	0.00	2	0.00	1.00
0.00	4	0.00	0.00	0.00	1.00	2	0.00	1.00
0.00	4	0.00	0.00	0.00	1.00	3	0.00	0.00
1.00	1	1.00	0.00	0.00	0.00	1	1.00	0.00
1.00	1	1.00	0.00	0.00	0.00	1	1.00	0.00
1.00	2	0.00	1.00	0.00	0.00	2	0.00	1.00
1.00	1	1.00	0.00	0.00	0.00	1	1.00	0.00
1.00	2	0.00	1.00	0.00	0.00	1	1.00	0.00
1.00	3	0.00	0.00	1.00	0.00	2	0.00	1.00
1.00	3	0.00	0.00	1.00	0.00	2	0.00	1.00
1.00	4	0.00	0.00	0.00	1.00	2	0.00	1.00

sal_grp_d_	Geographi	City	Distance	Working_s	shift_grp_	shift_grp_	shift_grp_	Hoursofwo
0.00	1		2	1	1.00	0.00	0.00	2
0.00	1		1	1	1.00	0.00	0.00	1
0.00	1		1	1	1.00	0.00	0.00	1
0.00	1		1	1	1.00	0.00	0.00	1
0.00	1		1	1	1.00	0.00	0.00	1
0.00	1		2	1	1.00	0.00	0.00	1
0.00	1		2	1	1.00	0.00	0.00	1
0.00	1		2	1	1.00	0.00	0.00	1
1.00	1		3	1	1.00	0.00	0.00	1
1.00	1		4	1	1.00	0.00	0.00	1
1.00	1		4	1	1.00	0.00	0.00	1
0.00	1		3	1	1.00	0.00	0.00	1
0.00	1		3	1	1.00	0.00	0.00	1
0.00	1	Alahsa	2	3	0.00	0.00	1.00	1
0.00	1	Ras tanura	3	1	1.00	0.00	0.00	1
0.00	1	Dammam	1	1	1.00	0.00	0.00	1
0.00	1	Bugaig	4	1	1.00	0.00	0.00	1
0.00	1	Bugaig	4	1	1.00	0.00	0.00	1
1.00	1	Ras tanura	2	1	1.00	0.00	0.00	1
0.00	1		1	1	1.00	0.00	0.00	1
1.00	1		2	1	1.00	0.00	0.00	1
0.00	1		1	1	1.00	0.00	0.00	1
1.00	1		4	1	1.00	0.00	0.00	1
1.00	1		3	1	1.00	0.00	0.00	1
1.00	1		3	1	1.00	0.00	0.00	1
1.00	1		2	1	1.00	0.00	0.00	1
1.00	1		2	1	1.00	0.00	0.00	1
1.00	1		2	1	1.00	0.00	0.00	1
1.00	1		1	1	1.00	0.00	0.00	1
0.00	1		1	1	1.00	0.00	0.00	1
1.00	1	Alkhobar	2	1	1.00	0.00	0.00	1
1.00	1	Alqatif	2	1	1.00	0.00	0.00	1
1.00	1	Alqatif	1	1	1.00	0.00	0.00	1
1.00	1	Alqatif	2	1	1.00	0.00	0.00	1
1.00	1		3	1	1.00	0.00	0.00	1
1.00	1	Alsafwa	2	1	1.00	0.00	0.00	1
1.00	1	Alsafwa	3	1	1.00	0.00	0.00	1
1.00	1	Alshargia	2	1	1.00	0.00	0.00	1
1.00	1	Alsafwa	2	1	1.00	0.00	0.00	1
1.00	1	Ras tanura	2	1	1.00	0.00	0.00	1
1.00	1	Ras tanura	3	1	1.00	0.00	0.00	1
1.00	1	Bogaig	4	3	0.00	0.00	1.00	1
1.00	1	Alkhobar	3	1	1.00	0.00	0.00	1
0.00	1	Dammam	3	1	1.00	0.00	0.00	1
0.00	1	Dammam	1	1	1.00	0.00	0.00	1
1.00	1	Dammam	2	1	1.00	0.00	0.00	1
1.00	1		2	1	1.00	0.00	0.00	1
0.00	1	Dammam	3	1	1.00	0.00	0.00	1
1.00	1		1	1	1.00	0.00	0.00	1

1.00	1		1	1	1.00	0.00	0.00	1
0.00	1		1	1	1.00	0.00	0.00	1
1.00	1	abqaiq	4	1	1.00	0.00	0.00	1
1.00	1	AbuMan	3	1	1.00	0.00	0.00	1
0.00	1	Bugaig	4	1	1.00	0.00	0.00	1
0.00	1	Bugaig	1	3	0.00	0.00	1.00	1
0.00	1	Dammam	1	1	1.00	0.00	0.00	1
0.00	1	Dammam	3	3	0.00	0.00	1.00	1
0.00	1	alrafiah	1	1	1.00	0.00	0.00	1
0.00	1		1	3	0.00	0.00	1.00	1
0.00	1		1	1	1.00	0.00	0.00	1
0.00	1		2	3	0.00	0.00	1.00	2
0.00	1		2	1	1.00	0.00	0.00	1
0.00	1	Alsafwa	2	3	0.00	0.00	1.00	1
0.00	1		3	1	1.00	0.00	0.00	1
0.00	1	Dammam	3	1	1.00	0.00	0.00	1
0.00	1	Dammam	3	1	1.00	0.00	0.00	1
0.00	1	Dammam	4	1	1.00	0.00	0.00	1
0.00	1	Ras tanura	3	1	1.00	0.00	0.00	1
0.00	1	Ras tanura	3	1	1.00	0.00	0.00	1
0.00	1		2	1	1.00	0.00	0.00	1
0.00	1		2	2	0.00	1.00	0.00	1
0.00	1		3	1	1.00	0.00	0.00	1
0.00	1		2	1	1.00	0.00	0.00	1
0.00	1		1	1	1.00	0.00	0.00	1
0.00	1		3	1	1.00	0.00	0.00	1
0.00	1	Alqatif	1	1	1.00	0.00	0.00	1
0.00	1	Alqatif	1	1	1.00	0.00	0.00	1
0.00	1	Alqatif	1	1	1.00	0.00	0.00	1
0.00	1	Alqatif	2	1	1.00	0.00	0.00	1
0.00	1	Bugaig	4	1	1.00	0.00	0.00	1
0.00	1	Alsafwa	3	1	1.00	0.00	0.00	1
0.00	1	Alsafwa	2	1	1.00	0.00	0.00	1
0.00	1	Alsafwa	1	1	1.00	0.00	0.00	1
0.00	1	Alsafwa	1	1	1.00	0.00	0.00	1
0.00	1	Alsafwa	1	1	1.00	0.00	0.00	1
0.00	1		4	1	1.00	0.00	0.00	1
0.00	1	Dammam	3	1	1.00	0.00	0.00	1
0.00	1	Dammam	4	1	1.00	0.00	0.00	1
0.00	1	Alsafwa	3	3	0.00	0.00	1.00	1
0.00	1	Ras tanura	1	1	1.00	0.00	0.00	1
0.00	1	Ras tanura	3	1	1.00	0.00	0.00	1
0.00	1	Ras tanura	2	1	1.00	0.00	0.00	1
0.00	1		4	1	1.00	0.00	0.00	1
0.00	1	Bugaig	4	1	1.00	0.00	0.00	1
0.00	1		4	1	1.00	0.00	0.00	1
0.00	1	Alkhobar	2	1	1.00	0.00	0.00	1
0.00	1	Dammam	2	1	1.00	0.00	0.00	1
0.00	1		3	1	1.00	0.00	0.00	1
0.00	1	Dammam	3	1	1.00	0.00	0.00	1

0.00	1 Dammam	2	1	1.00	0.00	0.00	1
0.00	1 Dammam	3	1	1.00	0.00	0.00	1
0.00	1 Dammam	3	1	1.00	0.00	0.00	1
0.00	1 Dammam	4	1	1.00	0.00	0.00	1
0.00	1 Alsafwa	1	1	1.00	0.00	0.00	1
0.00	1	4	1	1.00	0.00	0.00	1
0.00	1	2	1	1.00	0.00	0.00	1
0.00	1	2	1	1.00	0.00	0.00	1
0.00	1 Alqatif	1	1	1.00	0.00	0.00	1
0.00	1 Alqatif	1	1	1.00	0.00	0.00	1
0.00	1	2	1	1.00	0.00	0.00	1
0.00	1 Bugaig	4	1	1.00	0.00	0.00	1
0.00	1 Alsafwa	1	1	1.00	0.00	0.00	1
0.00	1 Um Alsahil	2	1	1.00	0.00	0.00	1
0.00	1 Alsafwa	2	1	1.00	0.00	0.00	1
0.00	1	1	1	1.00	0.00	0.00	1
1.00	1	1	2	0.00	1.00	0.00	1
1.00	1 Alqatif	2	1	1.00	0.00	0.00	1
0.00	1	2	1	1.00	0.00	0.00	1
0.00	1	1	1	1.00	0.00	0.00	1
0.00	1 Alsafwa	1	2	0.00	1.00	0.00	1
0.00	1 Dammam	3	1	1.00	0.00	0.00	1
0.00	1	1	1	1.00	0.00	0.00	1
0.00	1 Dammam	3	1	1.00	0.00	0.00	1
0.00	1	4	1	1.00	0.00	0.00	1
0.00	1 Dammam	1	1	1.00	0.00	0.00	1
1.00	1	1	1	1.00	0.00	0.00	1
0.00	1	2	1	1.00	0.00	0.00	1
1.00	1 Dammam	3	1	1.00	0.00	0.00	1
0.00	1 Dammam	2	1	1.00	0.00	0.00	1
0.00	1 Alrafya	1	1	1.00	0.00	0.00	1
0.00	1 Dammam	4	1	1.00	0.00	0.00	1
0.00	1 Alkhobar	3	1	1.00	0.00	0.00	1
0.00	1 Dammam	1	1	1.00	0.00	0.00	1
0.00	1 Dammam	1	3	0.00	0.00	1.00	1
1.00	1 Dammam	1	1	1.00	0.00	0.00	1
0.00	1 Saihat	1	1	1.00	0.00	0.00	1
0.00	1 Dammam	2	1	1.00	0.00	0.00	1
0.00	1 Dammam	2	1	1.00	0.00	0.00	1
0.00	1 Dammam	3	1	1.00	0.00	0.00	1
0.00	1 Dammam	2	1	1.00	0.00	0.00	1
0.00	1 Dammam	2	1	1.00	0.00	0.00	1
0.00	1	4	1	1.00	0.00	0.00	1
0.00	1 Alsafwa	2	1	1.00	0.00	0.00	1

hour_grp_(	hour_grp_(	Leave_job	Pay_dimer	Promotion	supervisior	Rewards_c	Procedures	Coworkers
0.00	1.00	2	8	10	19	4	8	11
1.00	0.00	2	4	8	22	3	6	10
1.00	0.00	1	2	6	10	2	4	15
1.00	0.00	2	10	10	22	5	10	12
1.00	0.00	2	8	7	15	4	8	9
1.00	0.00	2	9	7	12	2	7	9
1.00	0.00	2	4	6	17	4	4	11
1.00	0.00	2	8	9	22	4	6	11
1.00	0.00	2	5	7	20	4	8	11
1.00	0.00	2	7	6	20	4	6	12
1.00	0.00	2	6	5	22	4	6	14
1.00	0.00	2	6	8	17	4	8	13
1.00	0.00	1	6	7	15	3	4	7
1.00	0.00	1	4	3	12	1	4	11
1.00	0.00	2	5	6	15	4	7	10
1.00	0.00	1	6	5	17	4	8	10
1.00	0.00	1	9	8	15	3	6	7
1.00	0.00	2	7	8	18	4	8	8
1.00	0.00	2	8	8	14	3	6	9
1.00	0.00	1	4	2	6	1	2	3
1.00	0.00	1	7	7	17	4	8	8
1.00	0.00	1	7	6	15	4	4	10
1.00	0.00	1	7	8	18	4	7	12
1.00	0.00	2	9	8	20	3	6	9
1.00	0.00	1	3	4	12	3	3	10
1.00	0.00	1	8	8	18	4	6	10
1.00	0.00	1	4	2	11	3	4	8
1.00	0.00	2	2	6	9	1	2	13
1.00	0.00	1	4	4	16	3	6	11
1.00	0.00	2	5	6	14	3	6	8
1.00	0.00	2	7	7	22	4	8	10
1.00	0.00	2	7	8	13	3	8	9
1.00	0.00	1	4	5	8	2	4	8
1.00	0.00	1	6	8	15	2	3	10
1.00	0.00	1	3	4	18	3	7	14
1.00	0.00	2	6	7	17	2	6	11
1.00	0.00	1	7	7	16	3	4	10
1.00	0.00	1	3	7	19	4	4	9
1.00	0.00	2	8	8	24	5	10	13
1.00	0.00	2	9	10	22	4	5	12
1.00	0.00	2	8	8	23	4	10	7
1.00	0.00	2	8	8	18	5	6	11
1.00	0.00	2	8	8	20	4	8	10
1.00	0.00	1	7	6	19	3	6	13
1.00	0.00	2	3	5	12	2	4	8
1.00	0.00	1	5	7	10	1	4	13
1.00	0.00	1	5	6	10	1	3	9
1.00	0.00	2	5	6	18	2	3	9
1.00	0.00	1	7	8	12	2	4	10

1.00	0.00	1	5	5	5	1	2	5
1.00	0.00	2	7	7	21	4	8	11
1.00	0.00	1	7	6	14	2	6	8
1.00	0.00	2	8	8	13	3	4	11
1.00	0.00	2	6	7	20	4	9	11
1.00	0.00	1	4	6	10	2	5	7
1.00	0.00	1	3	3	14	2	4	10
1.00	0.00	1	3	4	16	3	5	11
1.00	0.00	2	8	10	21	4	9	9
1.00	0.00	2	10	10	25	5	10	12
1.00	0.00	2	8	7	14	3	5	9
0.00	1.00	1	2	3	10	1	5	11
1.00	0.00	2	6	8	23	5	8	9
1.00	0.00	1	6	8	11	3	6	5
1.00	0.00	2	3	4	18	2	7	9
1.00	0.00	2	7	2	21	4	6	9
1.00	0.00	1	6	6	15	3	6	9
1.00	0.00	2	6	6	17	3	7	10
1.00	0.00	2	8	8	20	4	8	10
1.00	0.00	2	7	5	18	3	7	11
1.00	0.00	2	6	5	21	5	10	13
1.00	0.00	2	6	10	19	5	7	6
1.00	0.00	2	6	6	17	3	6	11
1.00	0.00	2	5	8	20	4	8	13
1.00	0.00	2	7	6	15	4	4	8
1.00	0.00	2	6	7	17	5	8	10
1.00	0.00	2	7	3	8	2	7	4
1.00	0.00	1	2	5	14	4	6	13
1.00	0.00	2	5	5	19	4	8	8
1.00	0.00	2	6	10	19	3	6	7
1.00	0.00	1	6	6	13	1	6	8
1.00	0.00	1	4	3	19	4	6	7
1.00	0.00	2	9	10	23	5	10	12
1.00	0.00	1	9	6	18	3	4	8
1.00	0.00	2	7	8	18	3	8	8
1.00	0.00	2	8	10	19	2	7	10
1.00	0.00	1	6	3	14	3	5	8
1.00	0.00	2	8	6	15	3	5	9
1.00	0.00	1	7	6	15	3	6	9
1.00	0.00	1	5	5	9	2	5	5
1.00	0.00	2	7	5	13	2	4	7
1.00	0.00	1	8	5	17	4	7	9
1.00	0.00	2	8	5	15	2	8	9
1.00	0.00	1	4	9	13	2	8	8
1.00	0.00	2	6	5	20	4	8	12
1.00	0.00	1	8	6	10	3	4	9
1.00	0.00	2	10	10	15	5	6	9
1.00	0.00	1	2	3	12	3	3	6
1.00	0.00	1	7	4	10	3	3	5
1.00	0.00	1	8	5	10	1	4	7

1.00	0.00	2	4	4	16	3	8	8
1.00	0.00	1	4	3	17	4	4	10
1.00	0.00	1	8	3	10	2	6	4
1.00	0.00	1	6	4	14	3	5	9
1.00	0.00	2	8	5	19	4	9	6
1.00	0.00	2	8	3	20	4	7	10
1.00	0.00	2	8	8	19	3	7	11
1.00	0.00	1	6	6	17	5	6	7
1.00	0.00	1	4	3	17	3	6	10
1.00	0.00	1	8	5	12	3	4	8
1.00	0.00	2	6	8	20	4	8	10
1.00	0.00	2	7	7	23	3	10	11
1.00	0.00	2	5	4	17	4	8	11
1.00	0.00	2	8	5	16	2	7	10
1.00	0.00	2	7	5	17	3	8	9
1.00	0.00	1	7	8	14	2	4	9
1.00	0.00	1	10	10	25	5	10	7
1.00	0.00	1	6	6	18	4	7	10
1.00	0.00	1	8	5	7	1	3	7
1.00	0.00	1	7	4	13	4	5	11
1.00	0.00	2	4	3	7	1	8	7
1.00	0.00	2	7	4	15	2	4	10
1.00	0.00	1	3	6	15	2	8	13
1.00	0.00	1	8	4	18	4	7	12
1.00	0.00	2	7	5	24	4	10	10
1.00	0.00	1	9	10	14	2	8	8
1.00	0.00	1	6	8	17	4	8	10
1.00	0.00	1	5	6	17	3	6	11
1.00	0.00	1	8	4	11	1	5	9
1.00	0.00	2	6	6	20	4	8	10
1.00	0.00	2	8	7	24	5	10	11
1.00	0.00	1	2	2	7	1	5	7
1.00	0.00	1	3	2	19	4	7	13
1.00	0.00	2	6	4	20	4	8	13
1.00	0.00	2	6	9	20	5	10	7
1.00	0.00	2	6	3	12	2	3	6
1.00	0.00	2	4	4	10	2	4	8
1.00	0.00	2	6	5	21	5	9	9
1.00	0.00	2	8	7	20	4	7	11
1.00	0.00	1	6	4	9	1	5	10
1.00	0.00	2	4	4	11	2	3	7
1.00	0.00	1	3	3	11	2	2	6
1.00	0.00	2	9	5	22	4	9	11
1.00	0.00	1	6	3	11	1	2	6

Communic	Responsibi	Patient_di	Social_dim	Work_dim	Total_Satisfaction
3	6	16	9	18	112
5	10	15	7	19	109
4	10	13	2	6	74
5	10	19	10	19	132
4	8	14	5	14	96
4	10	12	9	19	100
4	8	16	8	21	103
4	8	16	7	16	111
4	8	15	8	17	107
4	8	9	6	19	101
4	10	11	7	21	110
4	8	20	8	18	114
2	8	14	4	16	86
3	6	10	7	15	76
2	8	13	6	16	92
4	8	14	6	17	99
5	10	14	6	14	97
3	7	15	8	17	103
4	9	15	5	13	94
3	8	10	3	7	49
3	8	16	6	16	100
4	8	12	9	15	94
5	9	13	8	16	107
3	6	12	6	15	97
2	5	9	8	18	77
3	7	13	5	13	95
1	7	10	3	12	65
1	6	8	2	12	62
2	9	12	4	15	86
1	8	11	3	13	78
4	7	15	7	18	109
2	8	14	6	15	93
2	6	12	4	6	61
2	7	12	4	10	79
1	9	16	9	18	102
4	8	10	5	13	89
3	7	11	4	11	83
5	7	12	5	12	87
5	10	19	9	20	131
4	8	16	6	19	115
5	10	18	10	17	120
3	9	17	6	17	108
3	9	14	8	17	109
4	10	15	7	19	109
2	8	11	4	13	72
4	10	11	6	12	83
4	9	10	4	10	71
1	10	9	5	15	83
2	7	14	2	12	80

1	9	6	2	6	47
4	10	16	9	20	117
3	7	14	6	15	88
3	7	12	4	13	86
5	10	15	8	20	115
3	8	16	6	18	85
3	8	15	5	18	85
4	7	13	4	16	86
5	10	19	10	20	125
5	10	19	9	21	136
2	8	13	8	17	94
4	5	5	4	11	61
5	8	18	8	17	115
2	8	15	4	15	83
3	9	6	8	22	91
4	9	15	8	19	104
3	6	12	6	15	87
4	9	13	8	15	98
4	8	15	8	18	111
4	7	12	6	17	97
4	8	14	8	17	111
4	7	13	6	17	100
3	6	11	6	17	92
4	9	16	8	20	115
3	10	6	3	14	80
4	8	13	6	19	103
4	8	13	6	14	76
4	8	14	4	16	90
4	8	13	6	15	95
3	7	13	6	16	96
4	8	16	3	9	80
5	9	16	7	17	97
5	10	16	9	22	131
3	9	13	4	15	92
4	8	16	7	19	106
4	8	16	7	22	113
4	8	10	5	13	79
4	7	12	6	15	90
3	6	12	6	13	86
1	10	9	5	14	70
3	9	12	6	15	83
4	8	15	8	18	103
4	8	16	5	18	98
4	6	15	9	12	90
4	8	16	8	20	111
1	7	2	2	11	63
2	8	16	8	19	108
2	8	12	4	11	66
2	9	13	5	15	76
2	6	10	4	12	69

4	8	12	7	18	92
4	8	10	5	14	83
5	10	10	6	13	77
1	5	12	6	16	81
5	10	17	5	20	108
4	8	14	8	16	102
3	7	16	6	17	105
3	7	12	8	15	92
4	7	11	4	17	86
4	8	16	6	17	91
4	8	16	8	20	112
5	10	17	8	20	121
4	8	12	8	18	99
4	7	13	8	21	101
3	9	14	6	18	99
2	8	14	7	18	93
5	10	20	10	21	133
4	8	15	6	16	100
2	7	12	4	9	65
3	8	13	7	16	91
4	7	12	7	13	73
4	8	15	6	16	91
5	10	14	7	14	97
4	7	15	6	19	104
5	10	17	10	20	122
4	8	16	9	16	104
4	7	14	8	14	100
3	6	12	7	16	92
2	9	13	2	12	76
4	8	15	7	17	105
4	7	16	9	19	120
1	5	8	2	9	49
4	8	16	5	15	96
4	9	3	7	20	98
5	10	14	5	16	107
4	6	10	5	15	72
3	6	7	4	12	64
4	7	17	8	19	110
4	8	16	7	18	110
2	5	8	3	8	61
2	6	2	2	10	53
1	8	6	3	5	50
5	7	16	8	21	117
3	9	14	2	12	69
